# Utilizing machine learning-based QSAR model to overcome standalone consensus docking limitation in beta-lactamase inhibitors screening: a proof-of-concept study

**DOI:** 10.1186/s13065-024-01324-x

**Published:** 2024-12-20

**Authors:** Thanet Pitakbut, Jennifer Munkert, Wenhui Xi, Yanjie Wei, Gregor Fuhrmann

**Affiliations:** 1 Department of Biology, Pharmaceutical Biology, Friedrich-Alexander-Universität Erlangen-Nürnberg, Staudtstr. 5, 91058 Erlangen, Germany; 2 FAU NeW – Research Center New Bioactive Compounds, Nikolaus-Fiebiger-Str. 10, 91058 Erlangen, Germany; 3 Shenzhen Key Laboratory of Intelligent Bioinformatics and Center for High - Performance Computing, Shenzhen Institute of Advanced Technology, Chinese Academy of Sciences, Shenzhen, 518055 China

**Keywords:** Molecular docking, Consensus docking, Random forest-based QSAR model, Beta-lactamase inhibitory screening

## Abstract

In virtual drug screening, consensus docking is a standard in-silico approach consisting of a combined result from optimized docking experiments, a minimum of two results combination. Therefore, consensus docking is subjected to a lower success rate than the best docking method due to its mathematical nature, an unavoidable limitation. This study aims to overcome this drawback via random forest, an ensemble machine learning model. First, in vitro beta-lactamase inhibitory screening was performed using an in-house chemical library. The in vitro results were later used as a validation. Consequently, we optimized docking protocols for AutoDock Vina and DOCK6 programs. With an appropriate scoring function, we found that DOCK6 could identify up to 70% of all active molecules, double the inappropriate. Further consensus analysis reduced the success rate to 50%. Simultaneously, a false positive rate was down to 16%, which was experimentally favorable for a drug search. Finally, we trained two quantitative structure-activity relationship (QSAR) models using logistic regression as a reference model and a random forest as a test model. After combining consensus docking results, random forest-based QSAR outperformed a logistic regression by restoring the success rate to 70% and maintaining a low false positive rate of around 21%. In conclusion, this study demonstrated the benefit of using a random forest (machine learning)-based QSAR model to overcome a standard consensus docking limitation in beta-lactamase inhibitor search as a proof-of-concept.

**Supplementary Information:**

The online version contains supplementary material available at 10.1186/s13065-024-01324-x.

## Highlights

An optimized DOCK6 scoring can maximize the success in identifying active molecules by up to 70%.

Consensus docking can significantly reduce the false positive rate in determining experimental bioactive molecules compared to the best docking.

Integrating a random forest-based QSAR model into a virtual screening workflow extends the limited success rate of consensus docking.

An in-house screening reveals the first-time report of three bio beta-lactamase inhibitors.

**Supplementary Information:**

The online version contains supplementary material available at 10.1186/s13065-024-01324-x.

## Graphical abstract

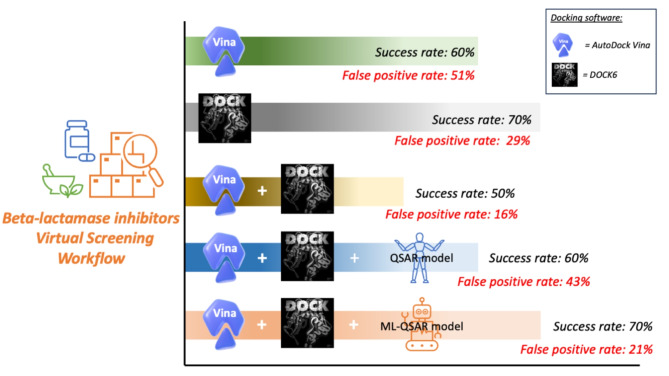

## Introduction

Since its introduction in the 1970s, computational-aided drug discovery and design (CADD) has continuously developed. A recent publication states that molecular docking is still among the most popular drug research tools used by leading academic laboratories and pharmaceutical companies, even though it was introduced nearly five decades ago [1]. Molecular docking belongs to a structural-based virtual screening (SBVS) approach [1]. Therefore, a 3D structure of the target protein with a known binding site is required to predict molecular interactions and binding affinity between the target and compounds of interest. Molecular docking has contributed to the success of multiple drug developments; for example, two recent anticancer drugs, Osimertinib and Venetoclax, were approved in 2017 and 2016 by the US FDA [2, 3]. However, molecular docking has a significant limitation regarding a low percentage of success rate and a high percentage of false positive rate, leading to undesirable accuracy [4]. This limitation only moderately affects conventional drug research since obtaining a few hit compounds from a virtual screening can generally satisfy the classical CADD in the early drug development phase. However, bacteria exhibit rapid drug resistance development. Therefore, this limitation can be a significant drawback. To address this issue, the docking protocol must be optimized to correspond to biological data and improve the docking accuracy, which we applied in this study. Additionally, the optimized docking protocol is the first step toward sustainable energy consumption for computation in the future, as public concern is rising [5].

Various approaches have been proposed for further optimization, along with the docking protocol [6]. A ligand-based model like a quantitative structure-activity relationship or QSAR model is one of the most commonly utilized approaches together with molecular docking to improve the computational search [6]. Unlike molecular docking, the QSAR model is a statistical model developed by molecular descriptors, like physio-chemical properties and/or molecular fingerprints obtained from active and inactive molecules [7]. According to recent studies, empowering the QSAR model with a machine-learning (ML) algorithm can improve computational drug discovery outcomes [6, 7]. Therefore, we aim to overcome the molecular docking challenge (low accuracy rate) by optimizing the docking protocol and combining it with the ML-QSAR model in this study.

Random forest (RF) is one of the most popular ML models [8] and is likely to outperform other models, such as support vector machines (SVM) and decision trees (DT) [9, 10]. RF is an ensemble ML model consisting of multiple sub-models [11]. This theoretically makes the RF prone to having fewer problems with model overfitting since it decides based on the average outcomes of its sub-models. Also, it leads to a more generalized model than other models like SVM and DT, which consist of one model [11]. Most importantly, RF can handle a high-dimensional dataset (extensive data features) [11], making RF more suitable for QSAR study. Therefore, the RF model was chosen to serve our purpose.

As mentioned earlier, the fast development of antimicrobial-resistant bacteria is one of the factors pressuring pharmaceutical research and development. It is commonly known that beta-lactamase is one of the common resistance mechanisms in bacteria and significantly contributes to an ongoing global health problem [12]. Therefore, we select beta-lactamase as our target to tackle the issue. Since 2022, we have invented a chemical library at the Division of Pharmaceutical Biology, Department of Biology, Faculty of Natural Sciences, Friedrich-Alexander-Universität Erlangen-Nürnberg, and it was named FARM-BIOMOL (**FA**U Pha**RM**aceutical biology-**BIO**active **MOL**ecules). This chemical library aims to provide an essential resource for initiating scientific research to fight against the bacterial antibiotic-resistant problem. Thus, FARM-BIOMOL was used in our study. Further information can be found on the website (https://pharmbio-fau-erlangen.github.io/FARM-BIOMOL/) [13].

**Fig. 1:**
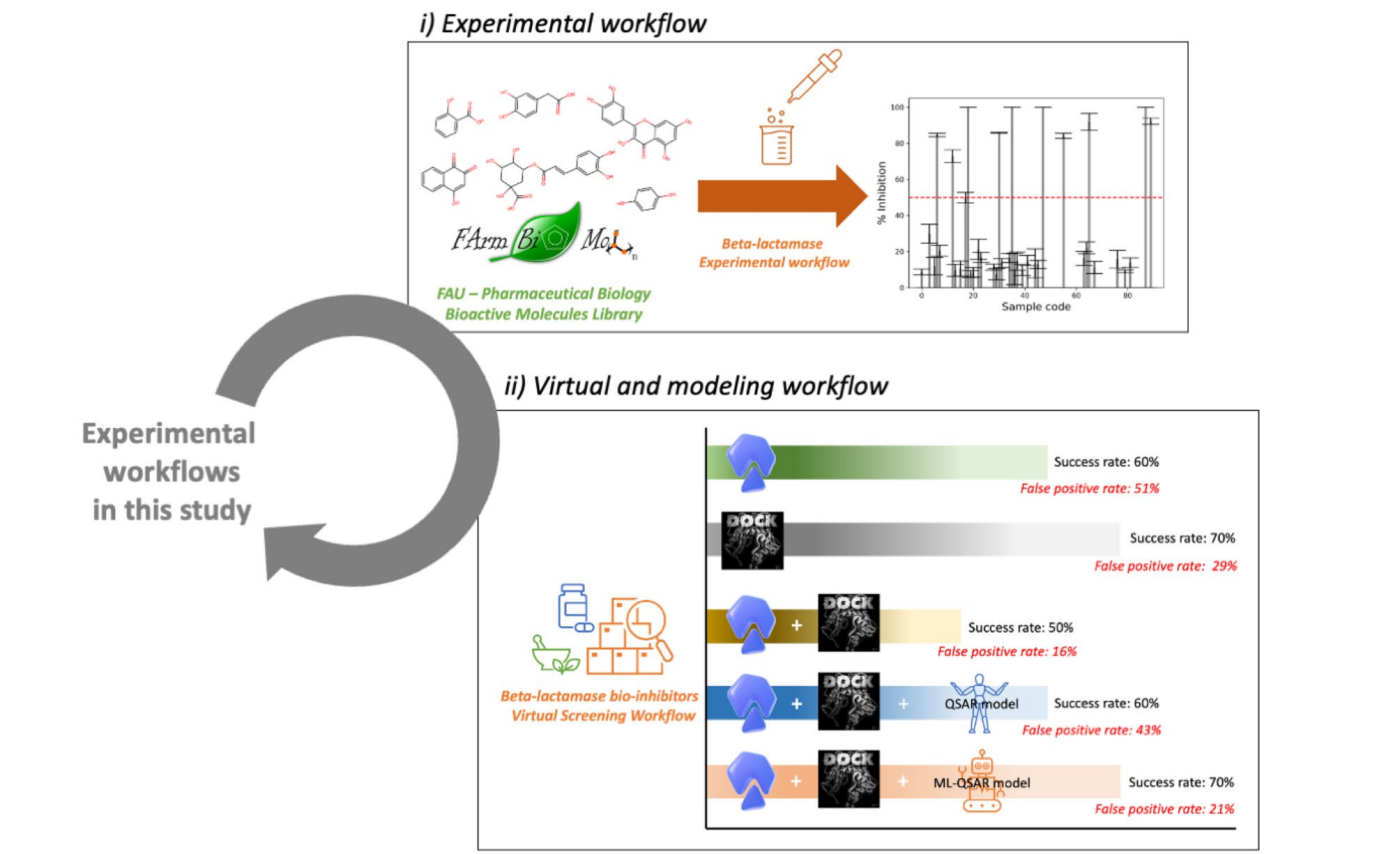
Graphical summary of two experimental workflows used in this study with main significant results. (i) represents an experimental workflow of an in vitro beta-lactamase inhibitory screening of a biomolecule from the FARMBIOMOL chemical library. The red dashed line indicates a cut-off criterion at 50% inhibition. (ii) represents virtual and modeling workflow from two molecular docking software (AutoDock Vina and DOCK6) and a QSAR model with and without machine learning. MS PowerPoint is used to generate this figure

As shown in Fig. 1, this study has two main workflows: (i) an in vitro enzyme-binding beta-lactamase inhibitory screening and (ii) the computational simulation and modeling. We screened eighty-nine compounds in our in-house chemical library, FARM-BIOMOL. The in vitro result was used as experimental validation data. Two standard molecular docking programs, AutoDock (AD) Vina [14] and DOCK6 [15], were used to perform virtual screening. The results from both dockings were cross-examined to evaluate a consensus docking. Finally, we constructed an RF-based QSAR model to improve consensus docking performance and used a logistic QSAR model as a baseline. Therefore, in this study, we provided a proof-of-concept of using the RF-based QSAR model to overcome the consensus docking challenge and improve virtual screening performance.

## Results

### In vitro enzyme-binding beta-lactamase inhibitory screening

**Fig. 2:**
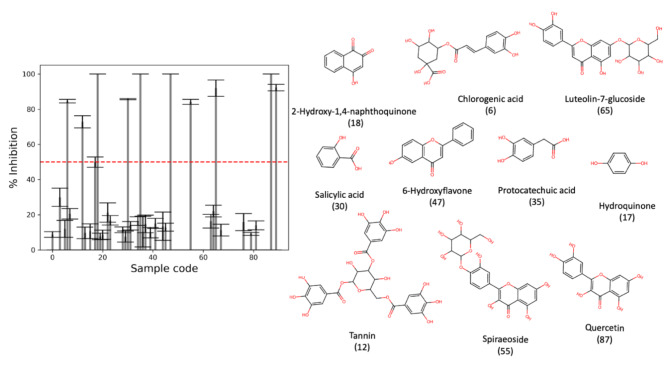
Anti-beta-lactamase inhibitory screening of eighty-nine compounds in the FARM-BIOMOL chemical library. The red dashed line indicates a selection criterion for an active inhibitory effect at 50% inhibition (Left). Ten active compounds pass the selection criteria (Right). All compounds are tested at the same concentration of 2 mg/ml. This figure is generated by Jupyter Notebook using Matplotlab and RDKit packages

Start with a brief introductory sentence saying what was done in these experiments. We first screened eighty-nine compounds from the library, and later, we found ten compounds demonstrating a promising positive effect against the beta-lactamase enzyme, showing an inhibitory activity of around and more than 50%, as shown in Fig. 2. We used potassium clavulanate as a clinical reference, exhibiting a 92 ± 1.88% inhibitory effect against the beta-lactamase enzyme (last column in Fig. 2, Left). Therefore, our screening is reliable based on the known positive control outcome. Notably, we used standard error in Fig. 2 (Left) due to a high deviation of less effective compounds due to a solubility problem. This problem is common in drug screening studies, especially when encountering natural products [16]. However, it is worth knowing that all active compounds’ standard deviation is in the 14–0% range, indicating a high reproducibility according to the previous report [17]. Complete screening data with standard deviation was provided in the supplementary file.

### Molecular docking and consensus docking

#### AD Vina

**Fig. 3:**
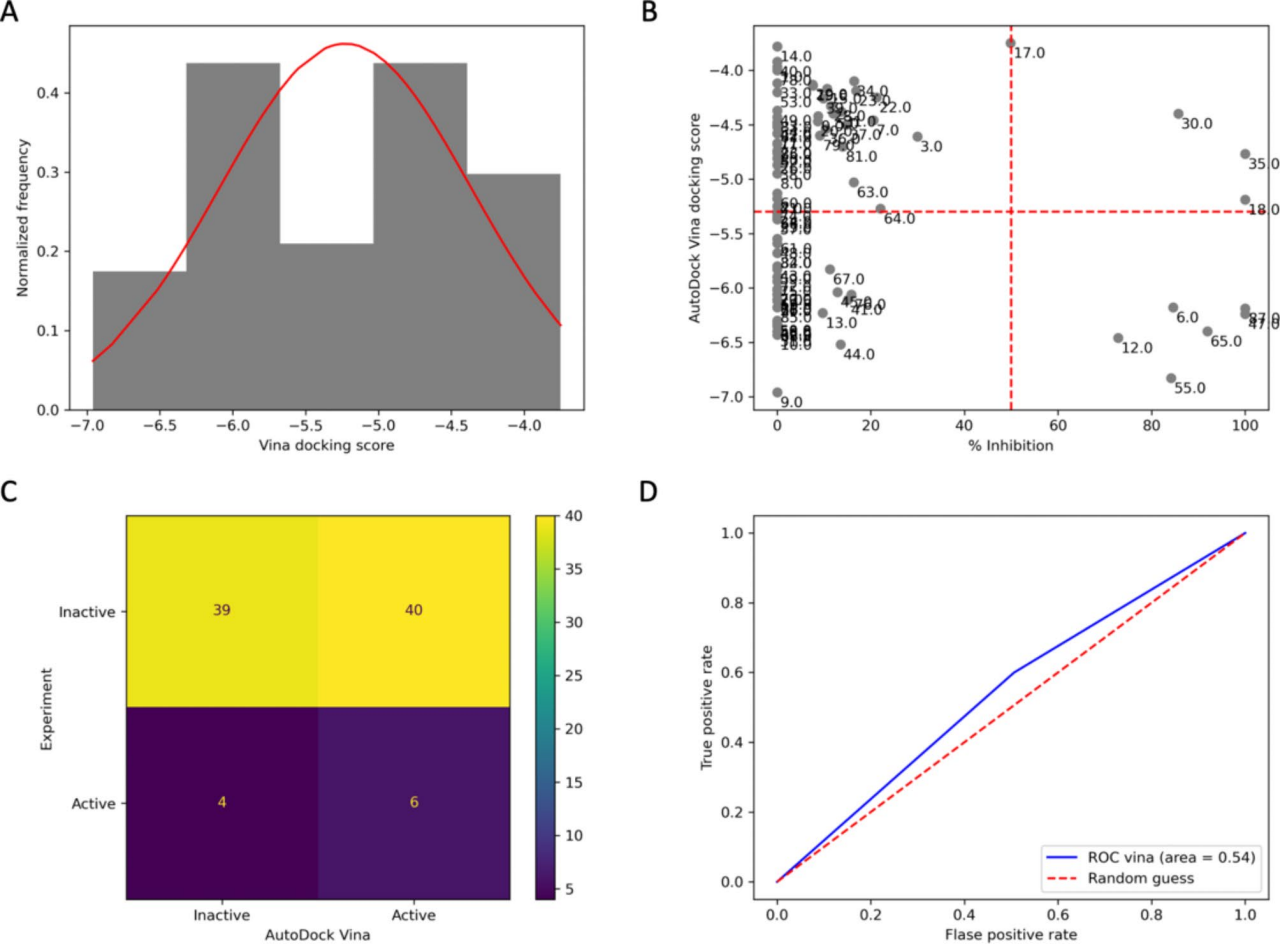
AD Vina’s predictability evaluation. (**A**) AD Vina’s results distribution. (**B**) A 2D scatter plot between AD Vina and experimental data. The red dashed line indicates a selection criterion at the average value of each axis. A label close to each point in the plot represents a compound number in the library. (**C**) A confusion matrix summarizes the 2D scatter plot. (**D**) AD Vina’s ROC curve and area. This figure is generated by Jupyter Notebook using the Matplotlab package

We validated the established docking protocol (described in the Methods section) through the redocking approach. The validation showed a satisfied outcome of the RMSD value of less than 3 Å. We provided a validation result in a supplementary file. Therefore, the result indicated a reliable outcome from our docking protocol. Later, this protocol was used to predict an anti-beta-lactamase activity of all eighty-nine compounds listed in the library. As a result, the average AD Vina docking score was − 5.23 ± 0.86 kcal/mol, the minimum score was − 3.75 kcal/mol, and the maximum score was − 6.96 kcal/mol. Figure 3A exhibited a normal distribution pattern of obtained results from AD Vina. Then, we used the 2D scatter plot between the AD Vina docking score and experimental data to evaluate the predictability of AD Vina. The half value of each axis was set as a cut-off, as shown in Fig. 3B. A confusion matrix was used to simplify the relationship between AD Vina and experimental data. Figure 3C demonstrated that AD Vina suggested forty-six compounds as active components against beta-lactamase using the cut-off value. Six of those components were true positive, experimentally exhibiting more than 50% inhibitory effect, while the other forty compounds were false positive. This led to a high false positive rate of nearly 51%. Finally, the author used a receiver operating characteristic curve and its area under the curve or ROC AUC score for an overall evaluation. The score of AD Vina was 0.54, which was slightly higher than a random guess score of 0.50. Even though AD Vina did not provide a satisfactory ROC AUC score, it could detect six out of ten experimental bioactive compounds. Therefore, AD Vina’s results were kept for further evaluation using a consensus docking approach.

**Table 1:** Overall performance of each docking model

	AD Vina	DOCK6gs	DOCK6des	Consensus docking
True Positive	6.00	3.00	7.00*	5.00
% True Negative	39.00	67.00*	56.00	66.00
% False Positive	40.00	12.00*	23.00	13.00
False Negative	4.00	7.00	3.00*	5.00
% Sensitivity (% True Positive Rate)	60.00	30.00	70.00*	50.00
% Specificity	49.37	84.81*	70.88	83.54
% False Positive Rate	50.63	15.19*	29.12	16.46
% Accuracy	50.56	78.65	70.79	79.78*
ROC_AUC	0.54	0.57	0.70*	0.66

Bold and asterisk indicate the best value

#### DOCK6

**Fig. 4:**
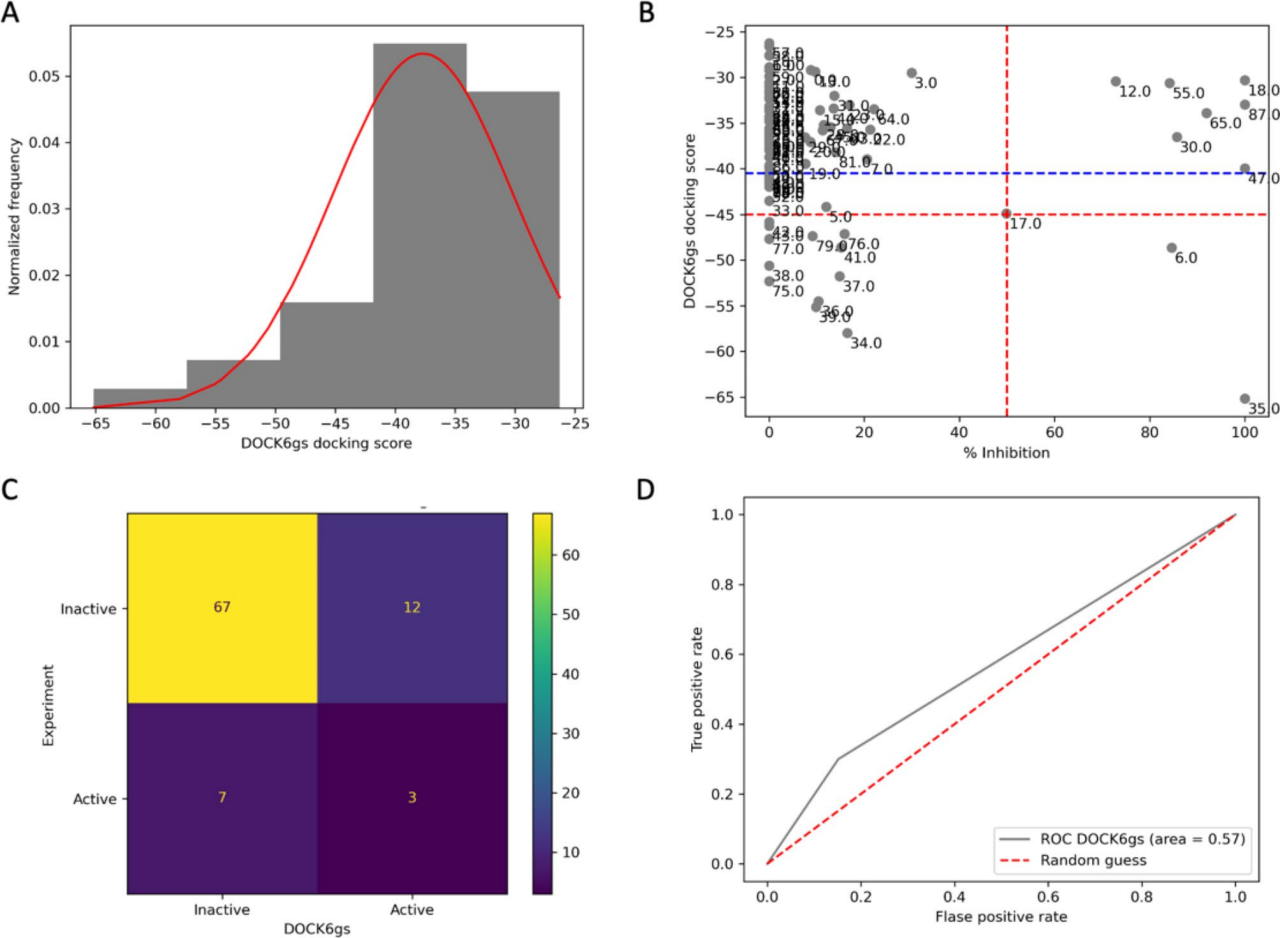
DOCK6gs’ predictability evaluation. (**A**) DOCK6gs results distribution. (**B**) A 2D scatter plot between DOCK6gs and experimental data. The red dashed line indicates a selection criterion at the average value of each axis. The blue dashed line is a new recentering criterion point after ignoring an outliner data of the y-axis (DOCK6gs scoring function). Points in the plot represent a compound number in the library. (**C**) A confusion matrix summarizes the 2D scatter plot. (**D**) DOCK6gs’ ROC curve and area. This figure is generated by Jupyter Notebook using the Matplotlab package

Unlike AD Vina, DOCK6 software offers various scoring functions to predict an enzyme-ligand binding affinity. Initially, we used a default function (grid search score). DOCK6 would be called DOCK6gs from this point onward. Like AD Vina, the established DOCK6gs docking protocol was validated and showed a satisfactory outcome before application. Further information on the validation result can be found in the supplementary information. As a result, the average DOCK6gs score was − 37.68 ± 7.47 kcal/mol, the minimum score was − 26.26 kcal/mol, and the maximum score was − 65.16 kcal/mol. The obtained DOCK6gs results did not show a normal distribution like AD Vina (Fig. 3A) but exhibited a stewed pattern toward a high score (Fig. 4A). Later, we constructed a 2D scatter plot using the same criteria as AD Vina. The graphical analysis showed only three experimental positive compounds were detected by DOCK6gs (Fig. 4B, red line). Since the distribution of the DOCK6gs was abnormal, it was rational to recenter the DOCK6gs docking score on the Y-axis by ignoring an outliner datapoint [18, 19]. Theoretically, it might recruit more of an actual positive candidate. Unfortunately, DOCK5gs results did not improve after recentering, as demonstrated in Fig. 4B, blue line. A confusion matrix (Fig. 4C) summarized a consequence from a scatter plot after recentering. Figure 4D exhibited a value of 0.57 from the AUC ROC score of DOCK6gs, which was slightly higher than AD Vina (0.54). Even though DOCK6gs’ true positive was only half of AD Vina, it showed a significantly lower false positive (high specificity). Therefore, the ROC AUC score of DOCK6gs was better than the AD Vina score. In conclusion, DOCK6gs’s result was unsatisfactory due to a low number of true positive candidates, determining three out of ten active compounds. Therefore, we changed a DOCK6gs docking score function to a descriptor function, which we provided in the following section.

**Fig. 5:**
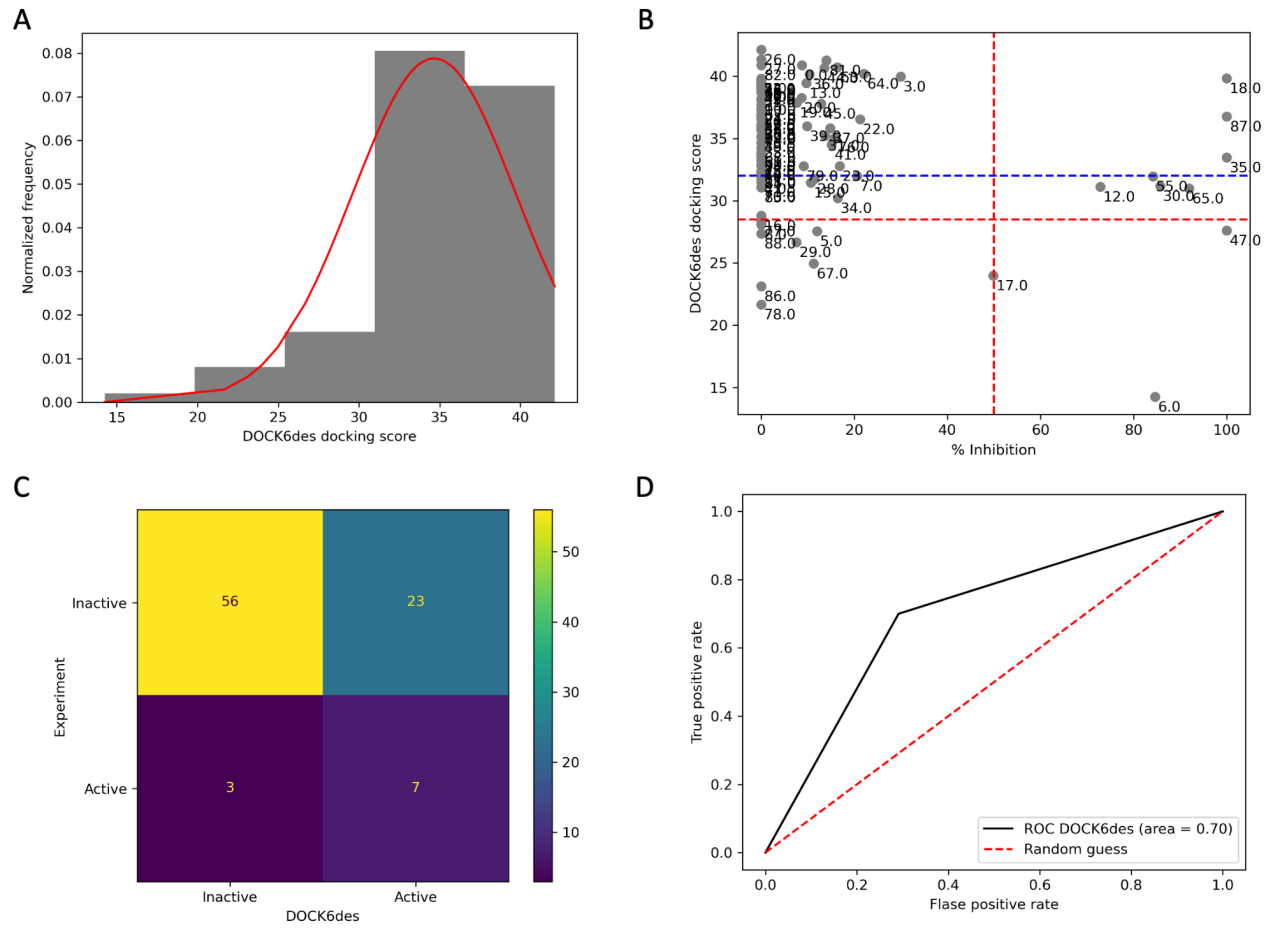
DOCK6des’ predictability evaluation. (**A**) DOCK6des results distribution. (**B**) A 2D scatter plot between DOCK6des and experimental data. The red dashed line indicates a selection criterion at the average value of each axis. The blue dashed line is a new recentering criterion point after ignoring an outliner data of the y-axis (DOCK6des scoring function). Points in the plot represent a compound number in the library. (**C**) A confusion matrix summarizes the 2D scatter plot. (**D**) DOCK6des’ ROC curve and area. This figure is generated by Jupyter Notebook using the Matplotlab package

To improve the result, we rescored DOCK6gs’ docking outcomes using another scoring function, a descriptor function, and it was named DOCK6des. A procedure similar to the one mentioned above was applied here. The average DOCK6des score was 34.66 ± 5.06 kcal/mol. The minimum DOCK6des score was 14.24 kcal/mol, and the maximum was 42.13 kcal/mol. The DOCK6des’ results exhibited a similar distribution pattern to DOCK6gs, as shown in Fig. 5A. Only three active components were detected in an initial evaluation (Fig. 5B, red line). However, after recentering the DOCK6des outcomes, four more active compounds could be included, as presented in Fig. 5B, blue line. Eventually, seven bioactive molecules were detected using a descriptor score function. A confusion matrix of DOCK6des was provided in Fig. 5C. The false positive rate of DOCK6des was around 29%, Table 1. The ROC AUC score of DOCK6des was 0.7 (Fig. 5D), which indicated a generally accepted discrimination power [20]. Since DOCK6des exhibited a satisfactory result, therefore, DOCK6des was chosen for further analysis.

#### Consensus docking

**Fig. 6:**
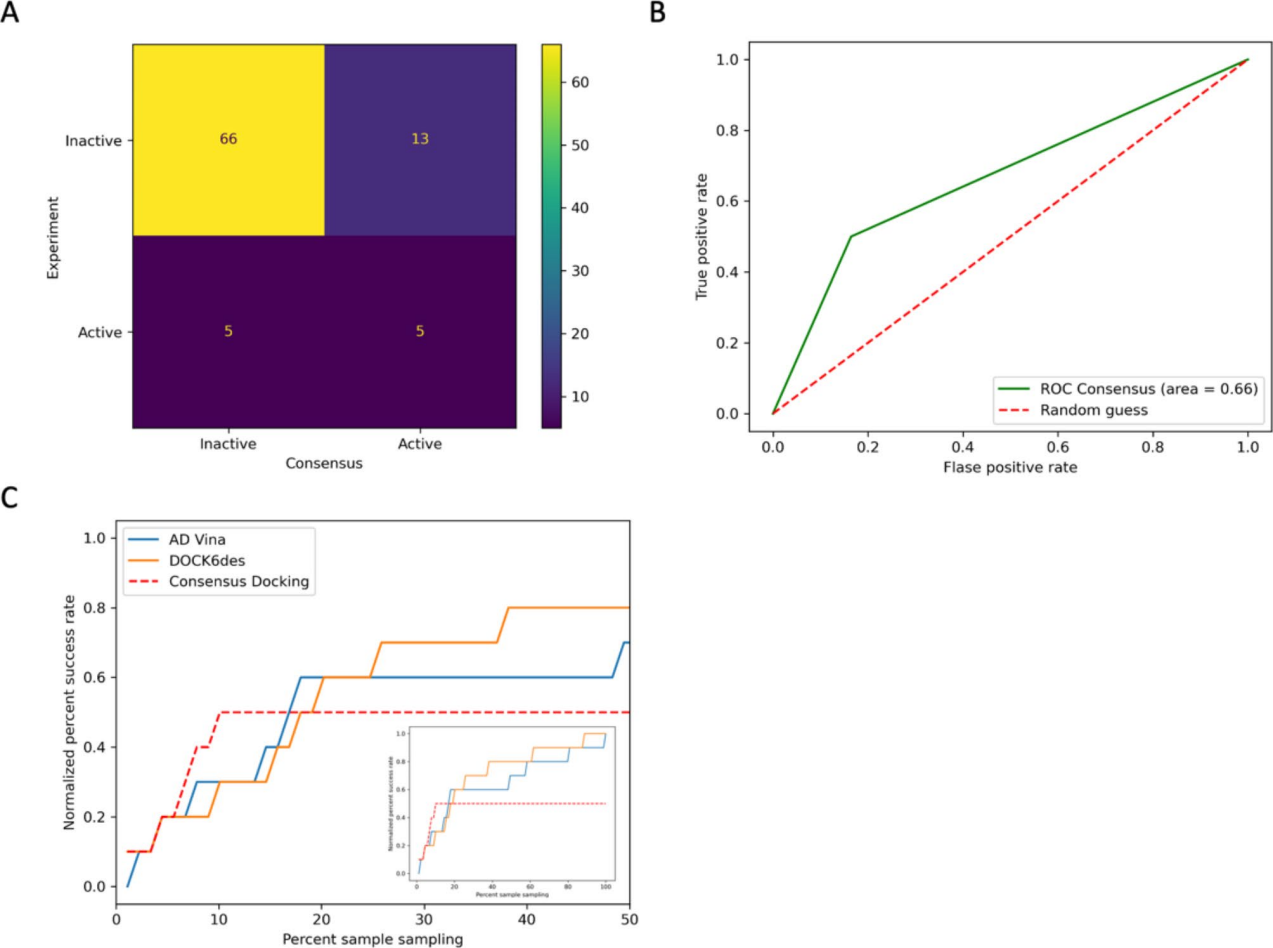
Consensus docking score predictability evaluation. (**A**) A confusion matrix of consensus docking. (**B**) consensus docking’s ROC curve and its area under the curve. (**C**) A cumulative success rate plot against a molecule sample size of each docking model up to 50% of the sample size from the chemical library. A total sample size result is shown in the small plot inside. This figure is generated by Jupyter Notebook using the Matplotlab package

Consensus docking has been widely applied in virtual screening studies to improve docking predictability [21]. The basic concept of consensus docking is that if a molecule shows a lower binding score in more than one docking method, it is likely that this molecule is experimentally active [22]. At least two docking results are analyzed to identify an overlap [21, 22]. Therefore, we cross-examined AD Vina’s and DOCK6des’ outcomes. Figure 6 exhibited the consensus docking result. Consensus docking detected only half of the bioactive molecules (five out of ten), less than AD Vina and DOCK6des, consequently detecting six and seven active substances. However, consensus docking significantly predicted lower false positives (only 13 molecules or 16% false positive rate) compared to AD Vina (40 molecules or nearly 51% false positive rate) and DOCK6des (23 molecules or around 29% false positive rate). These contributed to a higher accuracy rate of 79.78% from the consensus docking, as shown in Table 1. Still, consensus docking’s ROC AUC score of 0.66 (Fig. 6B) was slightly less than DOCK6des, which was 0.70 (Fig. 5D).

Next, we introduced the docking consensus score to prioritize consensus docking results by combining each molecule’s AD Vina and DOCK6des scores. A molecule with a lower score after the docking score combination was ranked as a higher priority than a higher score. Later, we plotted a cumulative success rate against tested molecule sample size from each docking method according to its score, as presented in Fig. 6C. Consensus docking outperformed AD Vina and DOCK6des predictability alone. With a consensus approach, testing only 10% of our chemical library (nine compounds) could detect 50% of the bioactive molecules. Five out of nine compounds predicted by consensus docking showed a strong inhibitory effect against beta-lactamase. Meanwhile, AD Vina and DOCK6des needed 17% (fifteen molecules) and 18% (sixteen molecules) to achieve the same level as consensus docking.

### Classification random forest (RF) descriptor-based QSAR model

#### Dataset recategorization

None of the docking approaches were able to detect all experimental bioactive molecules from the library. Docking could only detect three to seven active compounds depending on software and scoring function. To solve this problem, we applied a classification machine learning algorithm (random forest or RF) to construct a descriptor-based QSAR model. However, only ten out of eighty-nine molecules (around 11%) were considered active experimentally, as shown in Fig. 2. This led to a highly imbalanced dataset to train the ML model with an imbalance ratio of 7.9 when 1 is an ideal value, indicating a balanced dataset [23]. Training a model with a balanced dataset (active and inactive) is essential to maximize the model’s performance [24]. Therefore, we recategorized the experimental data into two categories to train the RF-based QSAR model more effectively. The first new category was a biologically observable group. All molecules that exhibited any inhibitory effect (% inhibition > 0) belonged to this group. The second group was non-biologically observable with % inhibition = 0. As a result, thirty-six compounds (36/89 or 39%) were re-grouped and defined as a new-active group (biologically observable group). This group was further categorized into two subgroups according to the potency. The first (weak) sub-group consisted of twenty-six weak inhibitors, and the second (strong sub-group) was ten robust inhibitors. The remaining fifty-three compounds (53/89 or 61%) were re-categorized as a new-inactive group (non-biologically observable group). In conclusion, data recategorization led to a more balanced dataset with an imbalance ratio of 1.5, allowing us to train the RF-based QSAR model effectively.

#### Training and testing set allocation

Nearly all data points from all categories, except a strong sub-group, were randomly split into training and testing sets using four-fold validation techniques to construct the RF model. The ten molecules from a strong inhibition sub-group were manually split. Five compounds detected by consensus docking were allocated to a training set. Three out of five molecules escaping docking were chosen for a training set. We manually picked compound numbers 30, 35, and 87 for the testing set. Compound number 35 could escape from AD Vina and DOCK6des, while AD Vina and DOCK6des could identify compound numbers 30 and 87 accordingly. Picking these three compounds into the testing set allowed us to evaluate the RF model performance corresponding to the docking method.

### RF descriptors-based QSAR model generation and evaluation

**Fig. 7:**
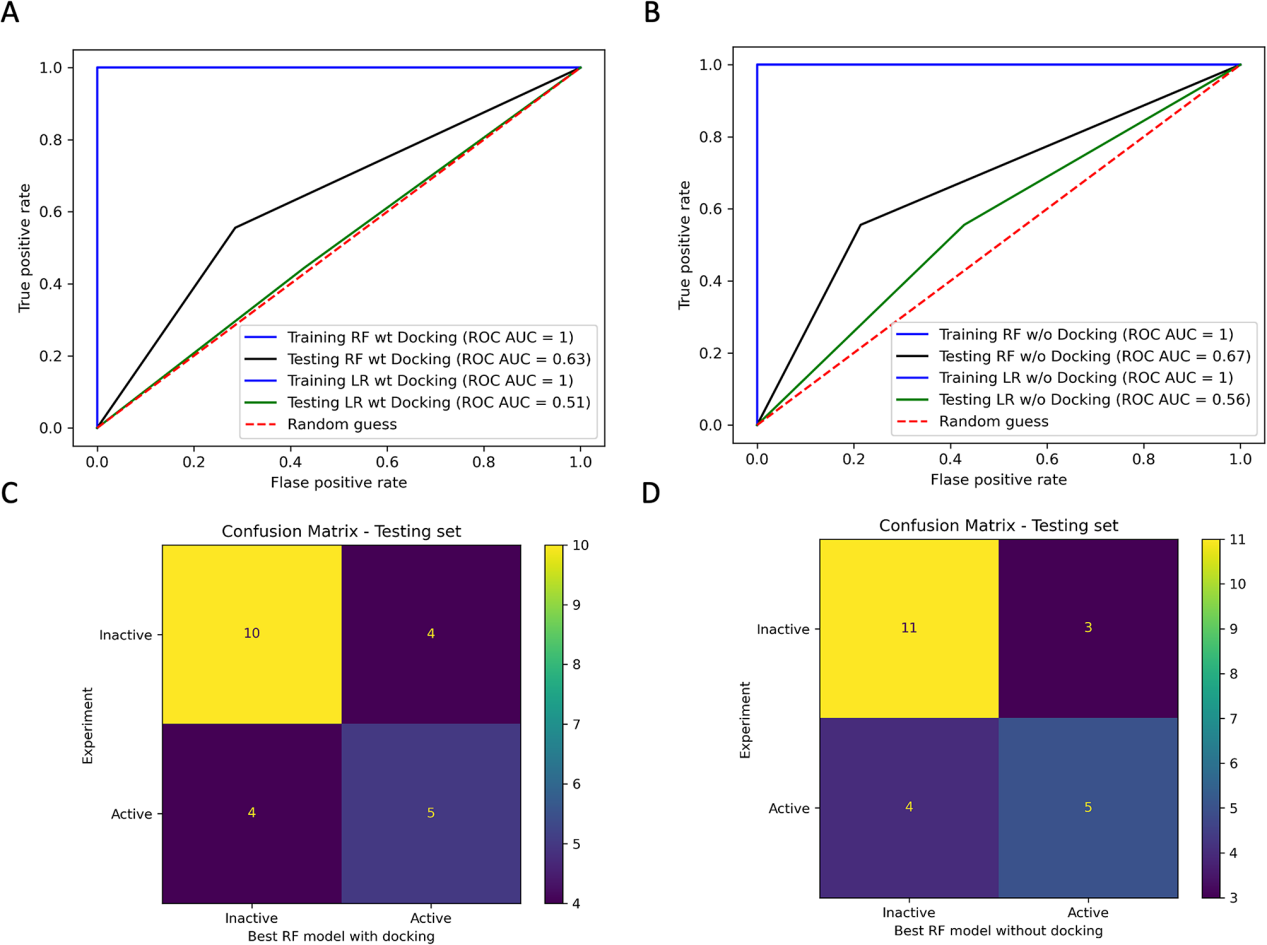
RF descriptors-based QSAR models predictability. (**A**) An ROC AUC score of the best RF model with consensus docking compares to the best LR model with consensus docking and a random guess. (**B**) An ROC AUC score of the best RF model without consensus docking compares to the best LR model with consensus docking and a random guess. (**C**) A confusion matrix of the testing set of the RF model with consensus docking. (**D**) A confusion matrix of the testing set of the RF model without consensus docking. This figure is generated by Jupyter Notebook using the Matplotlab package

After data scrubbing, 896 out of 1,875 descriptors from the PaDEL software remained. Information regarding these remaining descriptors is provided in the supplementary file. A binary consensus docking descriptor was added as an additional feature. Therefore, 897 descriptors in total were used to train RF-based QSAR models. Consequently, we generated thirty RF models by altering the random state parameter from 1 to 30 and used a logistic regression model as a baseline. A comprehensive evaluation of all created models can be found in the supplementary file. Five of those thirty generated models included an additional consensus docking descriptor in their models. The best RF model with consensus docking showed a false positive rate of 29% (Table 2) and an ROC AUC score of 0.63 from the testing set, Fig. 7A. On the other hand, the LR model’s false positive rate was nearly 43% (Table 2), and the ROC AUC score of 0.51 was slightly better than a random guess (the ROC AUC score of 0.50), Fig. 7A.

The remaining twenty-five RF models did not consider the consensus docking descriptor. The best RF model testing set without consensus docking exhibited a low % false positive rate of 21%, Table 2, and a slightly better ROC AUC score of 0.67, Fig. 7B, than the model with consensus docking. The LR model without consensus docking was also better than the model with docking. Still, the RF model outperformed the LR model here. We provided the overall performance of all models in Table 2.

**Table 2:** Overall performance of each model

	RF wt docking		LR wt docking		RF w/o docking		LR w/o docking	
	Training	Testing	Training	Testing	Training	Testing	Training	Testing
True Positive	27.00	5.00*	27.00	4.00	27.00	5.00*	27.00	5.00*
True Negative	39.00	10.00	39.00	8.00	39.00	11.00*	39.00	8.00
False Positive	0.00	4.00	0.00	6.00	0.00	3.00*	0.00	6.00
False Negative	0.00	4.00*	0.00	5.00	0.00	4.00*	0.00	4.00*
% Sensitivity (% True Positive Rate)	100.00	55.56*	100.00	44.44	100.00	55.56*	100.00	55.56*
% Specificity	100.00	71.43	100.00	57.14	100.00	78.57*	100.00	57.14
% False Positive Rate	0.00	28.57	0.00	42.86	0.00	21.43*	0.00	42.86
% Accuracy	100.00	65.22	100.00	52.17	100.00	69.57*	100.00	56.52
ROC_AUC	1.00	0.63	1.00	0.51	1.00	0.67*	1.00	0.56

Bold and asterisk indicate the best value of the testing sets of each model

Detailed analysis showed that both RF models could predict two molecules (compound 30 and 35) from a substantial inhibition sub-group but not compound 87. This analysis was provided in the supplementary file 1. In contrast, the LR models only predicted compound 30, which DOCK6 also detected. Noticeably, compound 87 was detected by AD Vina but not from both ML models. Therefore, it inferred that the AD Vina results had less impact on the ML model than DOCK6. Furthermore, compound 35 was able to escape all docking approaches. However, the RF model could predict compound 35 correctly. Therefore, this indicated the benefit that the RF model could compensate for the docking methods in bioactive molecule determination.

**Fig. 8:**
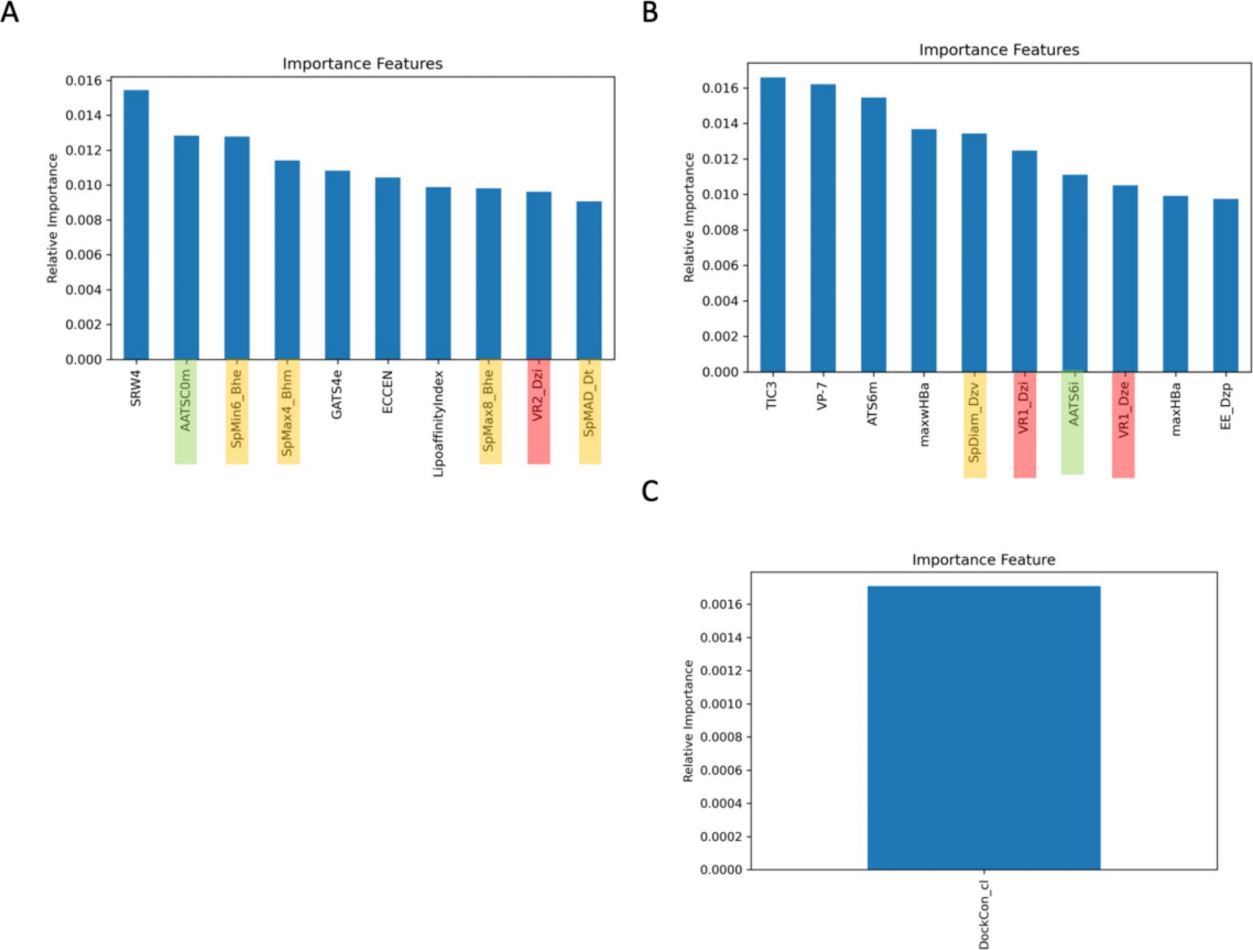
Top ten important features from RF models. (**A**) Important features from the RF model without docking consensus. (**B**) Important features from the RF model with consensus docking. (**C**) The relative importance of consensus docking as an important feature in the RF model. Highlighted colors indicate the same physiochemical property of descriptors as important features. This figure is generated by Jupyter Notebook using the Matplotlab package

Noticeably, some of the top ten important features of the models came from the same physio-chemical properties, as described in the following sentences and shown in Fig. 8. For example, AATSC0m and AATS61i were from the autocorrelation of the topological structure descriptor, and SpMin6_Bhe, SpMax4_Bhm, SpMaxB_Bhe, SpMAD_Dt, SpDiam_Dzv, VR2_Dzi, VR1_Dzi, and VR1_Dze were from the adjacency matrix descriptor. However, consensus docking was not listed among the top ten most important features, and the relatively important value of consensus docking was ten-fold less than that of TIC3 (the most important feature of the model).

## Discussion

Molecular docking plays an essential role in current drug discovery research [25]. Multiple docking programs offer various search algorithms to predict protein-ligand interactions, helping to identify new drug candidates [1, 25]. Therefore, optimizing the docking method before making a prediction is crucial to obtain the highest possible success rate. This study used two popular docking software (AD Vina and DOCK6) to optimize the docking search strategy for bioactive beta-lactamase inhibitors. We evaluated an in-house chemical library’s in vitro enzyme inhibition activity. The obtained experimental data was used as an experimental validation to cross-examine molecular docking and train ML-based QSAR models. As mentioned above, our result proved that combining the ML-based QSAR model with docking could surpass the standalone standard consensus docking challenge with a limited success rate.

Our screening experiment revealed the first-time report of the anti-beta-lactamase activity of two quinone metabolites (2-hydroxy-1,4-naphthoquinone or lawsone, compound 17, and hydroquinone, compound 18). Lawsone and hydroquinone are well-known plant secondary metabolites with wide-range biological potentials, from antioxidant and antimicrobial to anticancer properties [26–28]. Even in the current cosmeceutical market, a tropical product containing these metabolites is available, such as a natural hair dye product from *Lawsonia inermis* plant containing lawsone [26] and a prescribed depigmentation cream containing hydroquinone [29]. Still, our study has opened another potential application of lawsone and hydroquinone in inhibiting bacterial beta-lactamase activity against antibiotic-resistant bacteria. However, it is essential to note that exploring these compounds at a high concentration and/or for an extended period of time can cause toxicity [29, 30]. Therefore, utilizing a product containing these metabolites without supervision from a certified health provider is not recommended.

Furthermore, our screening also experimentally confirmed a predictive anti-beta-lactamase simulation of the previous study of polyphenol derivatives such as protocatechuic acid and tannin [31]. Unlike lawsone and hydroquinone, protocatechuic acid and tannin are typical natural products considered safe (GRAS) by the US Food and Drug Administration (FDA). According to the substances added to the food inventory list from the US FDA, they can be used in the food industry as flavoring and food additive agents. Therefore, developing an oral application from protocatechuic acid and tannin against bacterial beta-lactamase might be possible with further investigation.

Finally, our results agreed with earlier reports of known beta-lactamase inhibitors like salicylic acid [32], chlorogenic acid [33], quercetin, and its derivatives [34]. Interestingly, we obtained another promising activity from the flavonoid derivative, 6-hydroxyflavone, for the first time. To date, vast biological activities have been reported from polyphenols and flavonoids. Still, our study could reveal the potential of 6-hydroxyflavone for the first time, indicating that our understanding of these metabolic classes is incomplete. Therefore, further biological investigation should be continued to reveal the hidden pharmaceutical potentials of these metabolites.

In summary, this study reveals the first anti-beta-lactamase effect of three bioactive molecules, namely two quinones and one flavonoid. According to a recent study, only a fraction of beta-lactamase and inhibitor interactions were known [35]. Therefore, our screening experiment has filled the knowledge gap by revealing new beta-lactamase-inhibitor interactions. This will provide better data resources to construct more reliable ML and deep learning models for beta-lactamase search in the future.

In docking protocol optimization, multiple evaluation criteria can be used [36]. This study focused on three main criteria for a docking evaluation. The first and the most important is the success rate of experimental bioactive molecule identification. The second is a false positive rate, and the third is an overall docking model evaluation via ROC AUC score. First, the AD Vina protocol was optimized. AD Vina offers only one scoring function. Therefore, it is simple to optimize. As a result, AD Vina provided a satisfactory outcome with 60% accuracy in detecting bioactive compounds with a default scoring function. However, the false positive rate of AD Vina was unexpectedly high, around 51%. According to Weiss’ report, the false positive rate might be as high as 75% in a particular docking system [37]. This led to an unfavorable general performance of the model with a ROC AUC score of 0.54.

We further optimized the docking score function through the DOCK6 program to improve docking performance and found that the scoring function was essential in DOCK6 predictability. Applying a default grid score function minimized the success rate to as low as 30%. The success rate rose to 70% after changing the DOCK6 scoring function to the descriptor score. Furthermore, the false positive rate was also below 30%. As a result, DOCK6, with the descriptor score, generally performed well with a ROC AUC score of 0.70, indicating an accepted discrimination power. Luo and colleagues reported a similar finding from DOCK6 virtual screening for *Streptococcus mutans* sortase A inhibitor [38]. Luo’s study demonstrated that optimizing DOCK6 scoring functions improved discrimination power to nearly 90%, surpassing AutoDock’s performance of around 65% [38].

Later, we conducted a consensus docking analysis [21, 22]. Adopting the same evaluation criteria above, the performance of consensus docking was slightly less effective than the DOCK6 model. The success rate was reduced to 50%. However, the false positive was also down to 16%. Even though the error was reduced by nearly half, the consensus docking could not cope with a suppressed success rate, which led to a slightly lower general model performance (ROC AUC score = 0.66) than DOCK6 (ROC AUC score = 0.70). However, consensus docking naturally cannot outperform the best model since it identifies overlapping results from both dockings [21, 22]. The true benefit of consensus docking lies in the cumulative bioactive determination’s success rate. As presented in Fig. 6C, the plot showed that consensus docking required only the top 10% of the result to detect 50% of the experimental bioactive molecules. On the other hand, standalone docking methods require 17% as the minimum sample size to achieve the same success rate level as consensus docking. Our findings here aligned with Gupta’s experiments [39]. Gupta and coworkers revealed that covering the top 10% consensus docking result led to a 65% success rate in identifying active molecules against *Plasmodium falciparum* dihydrofolate reductase, while another docking approach like AD Vina and DOCK6 needed a larger sample size to reach the same level [39].

Even though the consensus docking approach increased the chance of bioactive identification and benefited the actual in vitro drug search, it also came with a great sacrifice. Half of the active compounds could escape this approach. Integrating a QSAR model with docking is one solution among the others that has been proposed to overcome this problem [6]. ML classification algorithms like RF have been reported to be more effective than other models [9]. Therefore, we selected RF to train a QSAR model. The result section showed that the RF-based QSAR model outperformed the classical LR-based QSAR model in all aspects. Most importantly, the RF models could detect two more bioactive compounds, one escaping all docking approaches. Therefore, combining consensus docking with the RF-based QSAR model extended the success rate by 20% in identifying bioactive molecules against beta-lactamase. The combined approach offered a success rate of 70% in total, equal to that of the best predictive model. Furthermore, the RF-based QSAR model maintained a low false positive rate of 21%, slightly higher than the lowest rate of 16%.

For a more comprehensive analysis, we compared our RF-based QSAR model performance to other existing models for beta-lactamase inhibitor search. However, we could only evaluate our model since other modes did not integrate docking. To this end, our RF classification models (with 0.67 ROC AUC score) generally performed better than a previous report by Anat and Gupta, with an ROC AUC score of nearly 51% [40]. However, Anant and Gupta’s model exhibited a 79% accuracy, which is more accurate than ours with almost 70%. Still, our accuracy score is better than that of another RF model by Papastergiou et al. [41], with an accuracy score of around 57%. According to our knowledge, Shi and colleagues reported the best RF-based QSAR model for beta-lactamase inhibitor virtual screening with a high ROC AUC of 0.88 and an accuracy score of 76% [42]. However, it is essential to note that Shi and colleagues used commercial software to generate descriptors and a much larger dataset (around a thousand compounds). On the other hand, we used an open-source program to generate physiochemical descriptors and smaller datasets (nearly a hundred compounds). Conn and coworkers demonstrated these factors contributed to the model’s performance [43]. Even if this is the case, our models provide a unique advantage over Shi’s model. Our model was trained using the results of one biological experiment, allowing the models’ predictions to be validated biologically easily. In contrast, Shi’s model used multiple results from various experimental conditions, leading to a practical problem in selecting a proper biological experiment to confirm a model prediction correctly.

To improve our model performance further, we plan to follow Shi’s study. Shi and colleagues showed that using SMILE (simplified molecular-input line-entry system) features and modifying descriptors through principle component analysis (PCA) could improve the model’s ROC AUC and accuracy scores [42]. Therefore, we plan to alter our current model to improve performance according to Shi’s report.

## Conclusion

In the current study, we proposed an alternative approach to overcome the consensus docking challenge by incorporating ML into the QSAR model. The obtained result indicated that an integrated ML-based QSAR model with an optimized docking protocol benefited a beta-lactamase inhibitor virtual screening by improving a success rate in bioactive molecule identification and maintaining a low false positive rate. Therefore, the current study laid an essential scientific background for further developing a combination of a virtual docking and ML-based QSAR model for beta-lactamase inhibitor search.

## Methods

### Chemicals and enzyme

We used an in-house chemical library, namely FARM-BIOMOL (**FA**U Pha**RMa**ceutical biology-**BIO**active **MOL**ecules) (https://pharmbio-fau-erlangen.github.io/FARM-BIOMOL/) [13] at the Division of Pharmaceutical Biology, Department of Biology, Faculty of Natural Sciences, Friedrich-Alexander-Universität Erlangen-Nürnberg, Erlangen, Germany. This library consists of eighty-nine compounds. Most of the compounds are natural products or their derivatives. The majority of the compounds were purchased commercially. However, some substances were self-isolated or synthesized in our laboratory. Beta-lactamase from *Bacillus cereus* 569/H9, nitrocefin, and potassium clavulanate were purchased from Sigma.

### In vitro enzyme-binding screening assays

The in vitro assay was modified from an existing protocol from the previous report [44]. We used 100 mM citrate buffer solution (pH 6) with 10 mM of ZnSO_4_.7H_2_O and 0.2% w/v NaN_3_ as a buffer solution. In the preparation step, all samples from the library were prepared at the same concentration of 4 mg/ml with a 4% DMSO as the maximum concentration for a sample stocking solution. For the assay, we tested the anti-beta-lactamase activity in a 96-well plate following the instructions below. First, 50 µl of buffer solution and 50 µl of 80x dilution from 1 mg/ml beta-lactamase solution (enzyme activity 75 mUnit/mg based on an in-house measurement, following a protocol from Sigma) were added to the wells. Later, the wells were mixed in 50 µl of 4 mg/ml sample solution or 0.4 mg/ml potassium clavulanate or 4% DMSO buffer solution. Then, 15 min of the incubation time at 37 degrees occurred. Finally, another 50 µl of 100x dilution of 1 mg/ml of nitrocefin (substrate) solution was added. The reaction was observed after 30 min by a microplate reader at 490 nm. As shown in the equation below, the percent inhibition was calculated as a fix-time measurement after sample color deduction. All tests were performed in triplicates.

$$\displaylines{\:\% \:inhibition = \: \cr \frac{{Absorptio{n_{control\:at\:30\:mins}} - Absorptio{n_{sample\:at\:30\:mins}}}}{{Absorptio{n_{control\:at\:30\:mins}}}} \cr \: \times \:\:100 \cr}$$

### Molecular docking preparation

#### Ligand preparation

The chemical information of all chemicals in the library was stored in SMILE format and obtained from the PubChem Database. Each compound’s 3D chemical structure was generated and energetically minimized using a general amber forcefield (GAFF) from Open Babel software (version 3.1.0) [45]. Following Zhu’s study, we used GAFF to optimize our compound of interest’s 3D chemical structures since GAFF provided a correlated prediction to experimental data [46]. Finally, Open Babel was used to prepare a proper format for docking simulation.

#### Beta-lactamase preparation

Beta-lactamase (PDB ID: 6F2N) [47] was downloaded from the RCSB PDB database and was used as a target enzyme for molecular docking since it was obtained from *B. cereus.* The same bacterial species produced beta-lactamase we used in our in vitro experiment. A native ligand that came with the protein crystal structure was used as a docking validation to ensure the reliability of the established docking protocols for AD Vina and DOCK6 before performing virtual screening.

#### Docking preparation and analysis

The Chimera program (version 1.17.3) [48] was utilized to prepare the beta-lactamase structure and necessary files for docking simulation for AD Vina [14] and DOCK6 [15] programs. The native ligand was used to navigate a catalytic domain, which was also set as a binding site for docking.

For AD Vina, the binding site was set as an x, y, and z coordination of 12.95 × 14.01 × 43.04 with a size of 15 cubic Å. We use a default value of nearly all AD Vina docking parameters, except exhaustiveness and number of docking poses. Exhaustiveness was adjusted to 128, and the number of docking poses was increased to 20. However, only the top 10 AD Vina predictive poses were used to calculate an average docking score. Finally, we selected AD Vina version 1.2.5 [14] to perform docking simulation.

For DOCK6, the author followed the user manual guide to set up the docking environment and used the sphgen_cpp package to generate the binding site. We used a fixed anchor docking option with a grid search parameter (a default scoring function). Later, we rescored DOCK6’s initial result using a descriptor scoring function.

Before performing virtual docking screening, all established docking protocols were validated by redocking the native ligand back into its original position. After passing the docking protocol validation, the result must be less than 3 Å than its original position [49]. Finally, for consensus docking, we identified overlapped active results from both docking through simple Linux commands (grep and diff).

### Classification ML models establishment and models evaluation

#### Data preparation

The physicochemical properties of chemicals listed in our’ library were generated via PaDEL software [50]. The software generated 1,875 descriptors in total. After data cleaning (removing constant value and quasi-constant feature, data with a low variance of less than 1%), only 896 descriptors remained. Furthermore, a binary consensus docking value was added as an additional descriptor. Then, the data was split into training and testing sets in a ratio of 3:1.

Imbalance ration estimation.

The imbalance ratio reported by Megahed et al. was used to evaluate the dataset’s balance [23]. The equation is provided below. A value of 1 indicates a balanced dataset and a higher value indicates a higher degree of imbalance.

$$\:Imbalance\:ratio=\:\frac{Number\:of\:negative\:samples}{Number\:of\:positive\:samples}\:$$

### RF and LR models establishment

We ran the Scikit-learn package [51] on Jupiter Notebook [52] to create RF and LR classification models. Initially, both models were established based on the default parameters. Except for the random number, the number was varied from 1 to 30 to generate 30 models for each ML. Later, in the hyperparameter tuning step, we applied the GridSearchCV approach, and grid parameters were set separately for each model. For the RF model, the grid parameters were set as bootstrap: True or False, max_depth: 10,30, 50, 70, 90, 100, 200, max_features; sqrt or log2, min_sample_leaf: 1, 2, 4, min_sample_split: 2, 5, 10, n_estimators: 50, 100, 200, 500, and criterion: gini or entropy. For the LR model, the grid parameters were set as solver: liblinear, sag or saga, penalty: l1 or l2, and C: 1, 10, 100, and 1000. Finally, we used fourfold cross-validation to assess the performance of both models.

### Model evaluation

We evaluated the model using an accuracy, confusion matrix, and ROC graph. Each evaluation parameter was calculated using the Scikit-learn package [51] running on the Jupiter Notebook [52].

## Electronic supplementary material

Below is the link to the electronic supplementary material.

Supplementary Material 1

## Data Availability

The current manuscript’s essential experiment data and relevant Python code can be found at https://github.com/ThanetPi/ML-QSAR-Docking-Proof-of-Concept.git.
