# Supplementary material for: Utilizing machine learning-based QSAR model to overcome standalone consensus docking limitation in beta-lactamase inhibitors screening: a proof-of-concept study
Source: BMC Chem. 2024 Dec 20;18(1):249. doi: 10.1186/s13065-024-01324-x (PMC11662792; doi:10.1186/s13065-024-01324-x)
Supplement: Supplementary file 1 — Supplementary Material 1 [file 13065_2024_1324_MOESM1_ESM.docx]

**Utilizing machine learning-based QSAR model to overcome standalone consensus docking limitation in beta-lactamase inhibitors screening: a proof-of-concept study.**

Thanet Pitakbut^1,3^, Jennifer Munkert^1,2^, Wenhui Xi^3^, Yanjie Wei^3^, Gregor Fuhrmann^1,2^

^1^ Friedrich-Alexander-Universität Erlangen-Nürnberg, Department of Biology,
 Pharmaceutical Biology, Staudtstr. 5, 91058 Erlangen, Germany

^2^ FAU NeW – Research Center New Bioactive Compounds, Nikolaus-Fiebiger-Str. 10, 91058 Erlangen, Germany

^2^ Shenzhen Key Laboratory of Intelligent Bioinformatics and Center for High
 Performance Computing, Shenzhen Institute of Advanced Technology, Chinese
 Academy of Sciences, Shenzhen 518055, China

Corresponding author: Gregor Fuhrmann (gregor.fuhrmann@fau.de)

**List of FigureS**

[**FigureS 1.** Anti-beta-lactamase inhibitory screening of eighty-nine compounds in the FARM-BIOMOL chemical library. The red dashed line indicates a selection criterion for an active inhibitory effect at 50% inhibition (Left). All compounds are tested at the same concentration of 2 mg/ml. The result is present in a format of mean ± SD. 3](#_Toc176683760)

[**FigureS 2.** AutoDock Vina docking protocol validation. Grey color represents beta-lactamase (PDB ID: 6F2N). Organce color indicates native ligands came with the crystal structure, and light blue color shows a re-docked native ligand that is back to its original position. The RMSD value of native and re-docked ligands is 2.769 Å. 4](#_Toc176683761)

[**FigureS 3.** DOCK6 docking protocol validation. Grey color represents beta-lactamase (PDB ID: 6F2N). Organce color indicates native ligands came with the crystal structure, and green color shows a re-docked native ligand that is back to its original position. The RMSD value of native and re-docked ligands is 0.807 Å. 5](#_Toc176683762)

[**FigureS 4.** Top ten important features from RF model 7, a non-selected model with the same best performance as RF model 1 used in the manuscript. For information can be found in TableS 3 below. 6](#_Toc176683763)

**List of TableS**

[**TableS 1.** Anti-beta-lactamase inhibitory screening of eighty-nine compounds in the FARM-BIOMOL chemical library. 6](#_Toc176683377)

[**TableS 2.** A list of descriptors used to train the machine learning-based QSAR model, excluding additional docking parameters. 9](#_Toc176683378)

[**TableS 3.** Random Forest models evaluation. 14](#_Toc176683379)

[**TableS 4.** Logistic regression models evaluation. 15](#_Toc176683380)


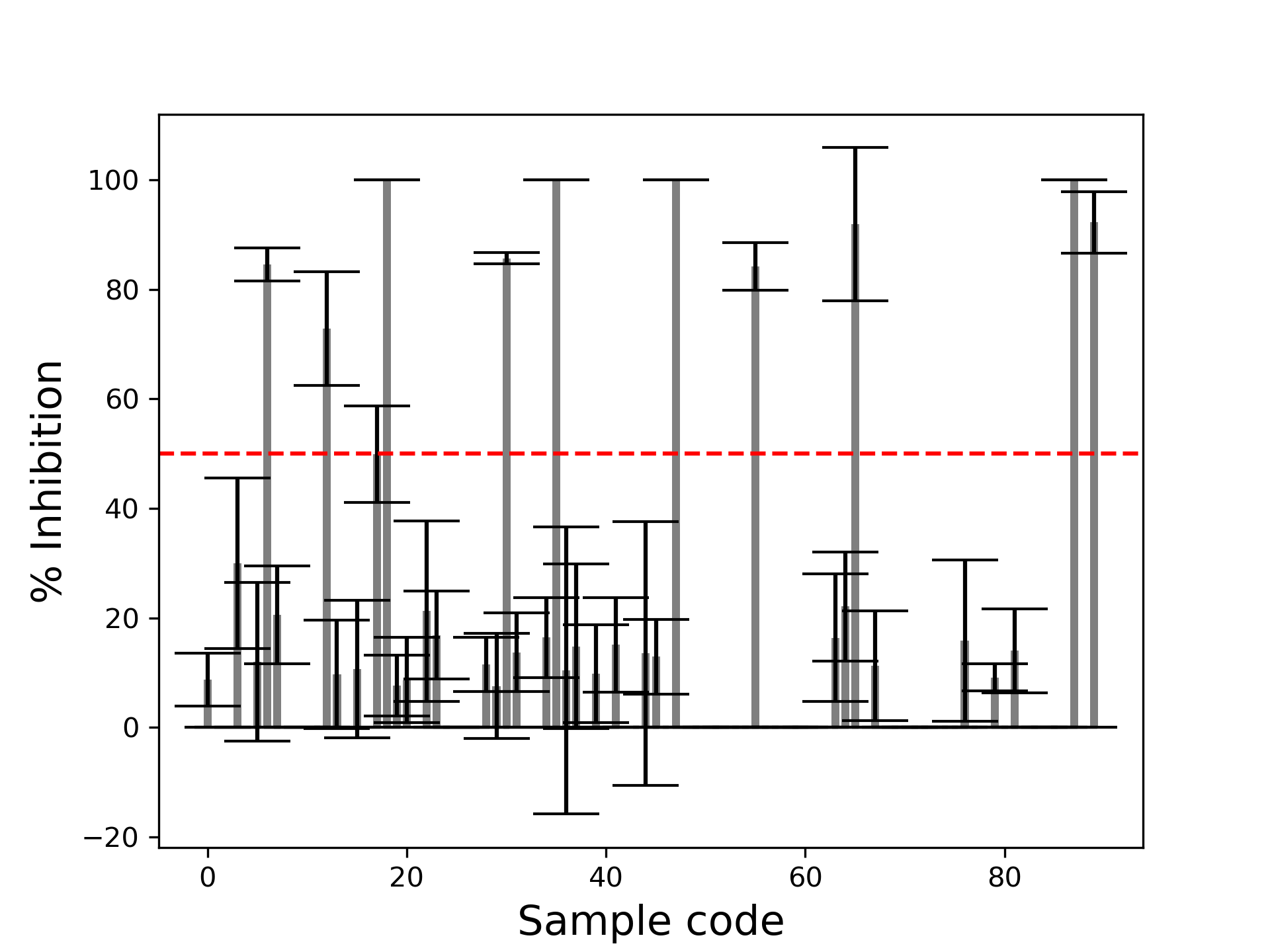


**FigureS 1.** Anti-beta-lactamase inhibitory screening of eighty-nine compounds in the FARM-BIOMOL chemical library. The red dashed line indicates a selection criterion for an active inhibitory effect at 50% inhibition (Left). All compounds are tested at the same concentration of 2 mg/ml. The result is present in a format of mean ± SD.


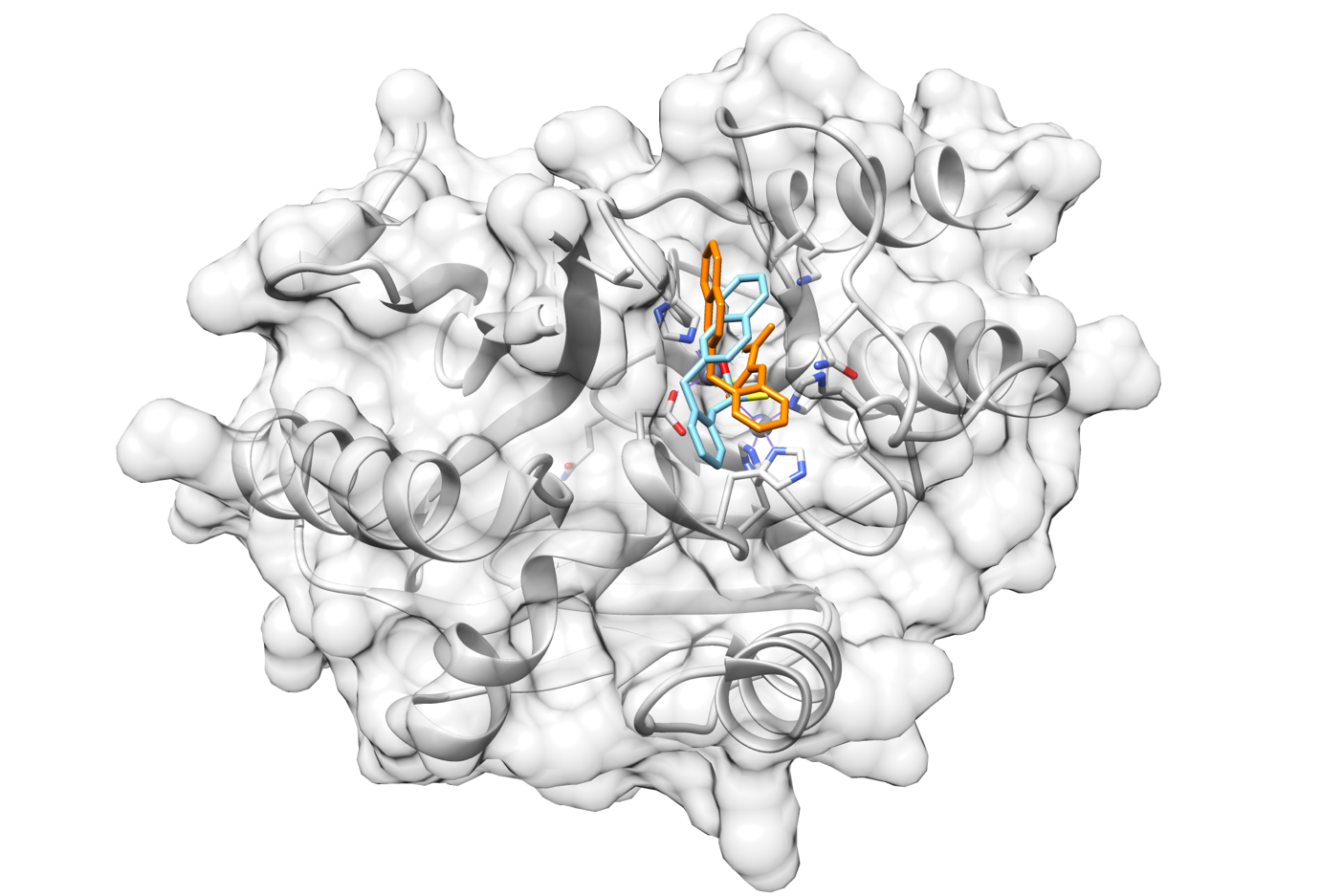


**FigureS 2.** AutoDock Vina docking protocol validation. Grey color represents beta-lactamase (PDB ID: 6F2N). Organce color indicates native ligands came with the crystal structure, and light blue color shows a re-docked native ligand that is back to its original position. The RMSD value of native and re-docked ligands is 2.769 Å.


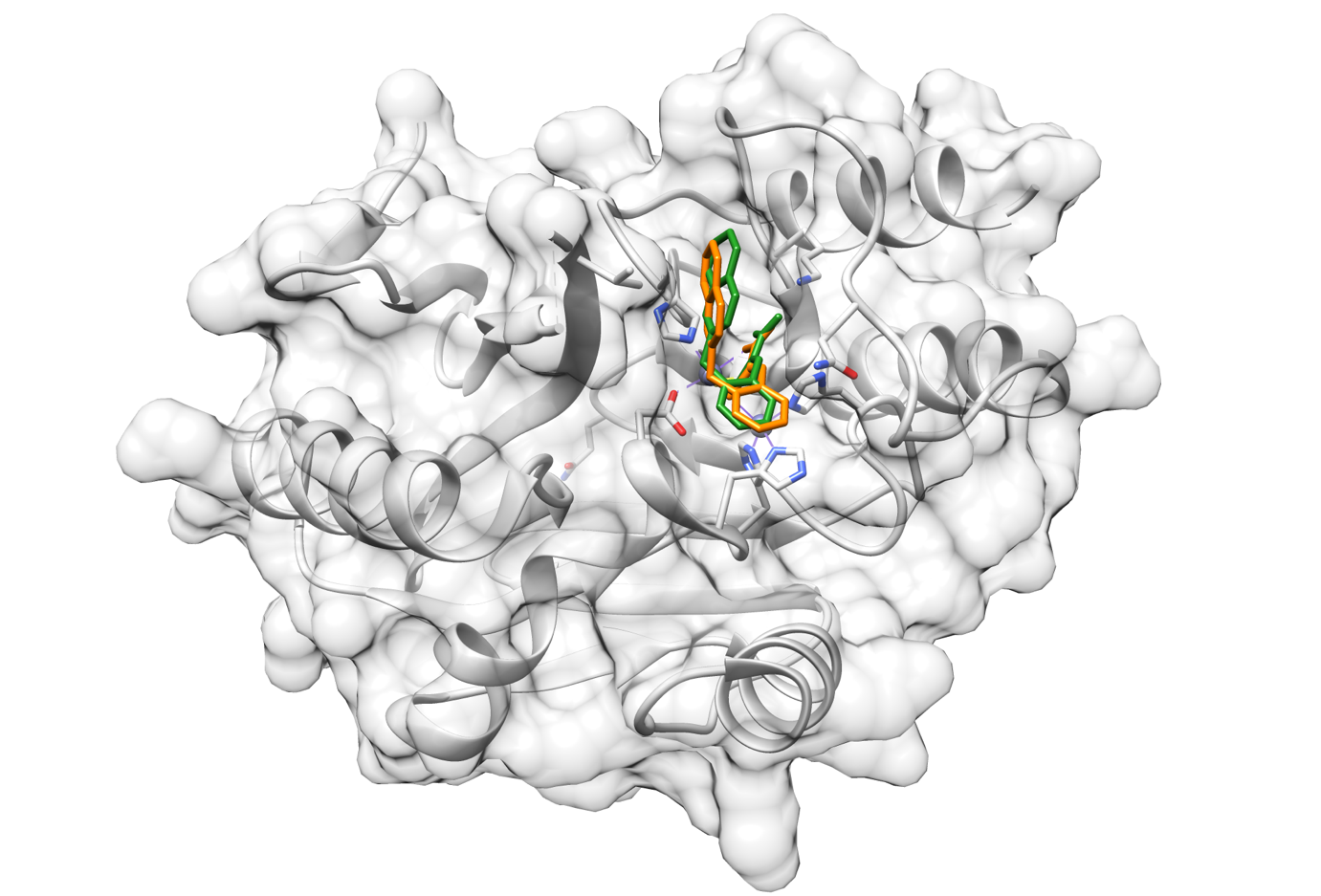


**FigureS 3.** DOCK6 docking protocol validation. Grey color represents beta-lactamase (PDB ID: 6F2N). Organce color indicates native ligands came with the crystal structure, and green color shows a re-docked native ligand that is back to its original position. The RMSD value of native and re-docked ligands is 0.807 Å.


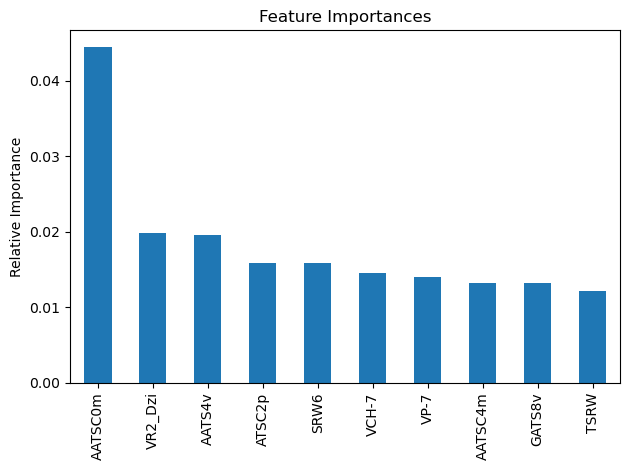


**FigureS 4.** Top ten important features from RF model 7, a non-selected model with the same best performance as RF model 1 used in the manuscript. For information can be found in TableS 3 below.

**TableS 1.** Anti-beta-lactamase inhibitory screening of eighty-nine compounds in the FARM-BIOMOL chemical library.

| **No** | **FARMBIOMOL ID.** | **% inhibition** | **SD** | **SE** | **Name** |
| --- | --- | --- | --- | --- | --- |
| 0 | 1 | 8,74 | 4,85 | 1,62 | thymol |
| 1 | 4 | 0,00 | 0,00 | 0,00 | catechol |
| 2 | 5 | 0,00 | 0,00 | 0,00 | carmine |
| 3 | 6 | 29,93 | 15,57 | 5,19 | coumarin |
| 4 | 7 | 0,00 | 0,00 | 0,00 | quinaldic acid |
| 5 | 8 | 12,02 | 14,49 | 4,83 | Trans-cinnamic acid |
| 6 | 9 | 84,57 | 2,98 | 0,99 | chlorogenic acid |
| 7 | 12 | 20,57 | 8,89 | 2,96 | caffeine anhydrous |
| 8 | 13 | 0,00 | 0,00 | 0,00 | caffeic acid |
| 9 | 15 | 0,00 | 0,00 | 0,00 | ellagic acid |
| 10 | 16 | 0,00 | 0,00 | 0,00 | esculin |
| 11 | 17 | 0,00 | 0,00 | 0,00 | ferulic acid |
| 12 | 18 | 72,87 | 10,39 | 3,46 | tannine |
| 13 | 19 | 9,70 | 9,89 | 3,30 | hesperidin |
| 14 | 20 | 0,00 | 0,00 | 0,00 | hydroquinone monomethyl ether |
| 15 | 21 | 10,68 | 12,58 | 4,19 | 4-hydroxylbenzoic acid |
| 16 | 22 | 0,00 | 0,00 | 0,00 | 2'-hydroxylflavone |
| 17 | 23 | 49,87 | 8,80 | 2,93 | hydroquinone |
| 18 | 24 | 100,00 | 0,00 | 0,00 | 2-hydroxy-1,4-naphtoquinone |
| 19 | 25 | 7,66 | 5,53 | 1,84 | methyl-4-hydroxybenzoate |
| 20 | 27 | 8,64 | 7,80 | 2,60 | m-Hydroxy-benzoic acid |
| 21 | 28 | 0,00 | 0,00 | 0,00 | Khellin |
| 22 | 29 | 21,25 | 16,49 | 5,50 | Kojic acid |
| 23 | 32 | 16,86 | 8,02 | 2,67 | Methyl 2-Methoxybenzoate |
| 24 | 33 | 0,00 | 0,00 | 0,00 | Nalidixic acid |
| 25 | 34 | 0,00 | 0,00 | 0,00 | Narcotine Hydrochloride |
| 26 | 35 | 0,00 | 0,00 | 0,00 | umbelliferone |
| 27 | 36 | 0,00 | 0,00 | 0,00 | Vitexin |
| 28 | 37 | 11,48 | 4,96 | 1,65 | Pyrogallo |
| 29 | 39 | 7,58 | 9,61 | 3,20 | Phloroglucinol dihydrate |
| 30 | 40 | 85,68 | 1,02 | 0,34 | Salcylic acid |
| 31 | 41 | 13,71 | 7,19 | 2,40 | salicylic acid methyl ester |
| 32 | 42 | 0,00 | 0,00 | 0,00 | D-salicin |
| 33 | 43 | 0,00 | 0,00 | 0,00 | alpha-terpinene |
| 34 | 44 | 16,41 | 7,34 | 2,45 | vanillin |
| 35 | 49 | 100,00 | 0,00 | 0,00 | Homoprotocatechuic acid |
| 36 | 50 | 10,40 | 26,21 | 8,74 | p-Hydroxyphenylpropionic acid |
| 37 | 51 | 14,82 | 15,04 | 5,01 | m-Hydroxyphenylacetic acid |
| 38 | 52 | 0,00 | 0,00 | 0,00 | Isoscopoletin |
| 39 | 54 | 9,84 | 8,96 | 2,99 | Nicotinamide |
| 40 | 55 | 0,00 | 0,00 | 0,00 | Nicotinic acid |
| 41 | 56 | 15,12 | 8,64 | 2,88 | Rutin |
| 42 | 58 | 0,00 | 0,00 | 0,00 | Eugenol |
| 43 | 61 | 0,00 | 0,00 | 0,00 | papaverine |
| 44 | 64 | 13,52 | 24,08 | 8,03 | rhein |
| 45 | 65 | 12,91 | 6,78 | 2,26 | aloin |
| 46 | 69 | 0,00 | 0,00 | 0,00 | emodin |
| 47 | 74 | 100,00 | 0,00 | 0,00 | 6-hydroxyflavone |
| 48 | 76 | 0,00 | 0,00 | 0,00 | atropamine |
| 49 | 77 | 0,00 | 0,00 | 0,00 | nicotine |
| 50 | 78 | 0,00 | 0,00 | 0,00 | L-Hyoscamine |
| 51 | 84 | 0,00 | 0,00 | 0,00 | Aloe-emodin |
| 52 | 86 | 0,00 | 0,00 | 0,00 | capsaicin |
| 53 | 88 | 0,00 | 0,00 | 0,00 | thujone |
| 54 | 89 | 0,00 | 0,00 | 0,00 | 1(s)-borneol |
| 55 | 97 | 84,17 | 4,33 | 1,44 | spiraeoside |
| 56 | 100 | 0,00 | 0,00 | 0,00 | chinchonine-hydrochloride |
| 57 | 101 | 0,00 | 0,00 | 0,00 | psoralen |
| 58 | 103 | 0,00 | 0,00 | 0,00 | Prunin |
| 59 | 104 | 0,00 | 0,00 | 0,00 | Santonin |
| 60 | 105 | 0,00 | 0,00 | 0,00 | Isopimpinellin |
| 61 | 106 | 0,00 | 0,00 | 0,00 | Lobeline Hydrochloride |
| 62 | 110 | 0,00 | 0,00 | 0,00 | Apigenin-7-glucoside |
| 63 | 111 | 16,35 | 11,64 | 3,88 | Isoferulic Acid |
| 64 | 112 | 22,08 | 9,97 | 3,32 | p-Arbutin |
| 65 | 113 | 91,93 | 13,98 | 4,66 | Luteolin-7-Glucoside |
| 66 | 114 | 0,00 | 0,00 | 0,00 | r-Atropine |
| 67 | 115 | 11,26 | 10,03 | 3,34 | Eserine/Physostigmine |
| 68 | 116 | 0,00 | 0,00 | 0,00 | Bergapten |
| 69 | 117 | 0,00 | 0,00 | 0,00 | Formononetin |
| 70 | 119 | 0,00 | 0,00 | 0,00 | Genistein |
| 71 | 120 | 0,00 | 0,00 | 0,00 | Belladonna total alkaloids |
| 72 | 121 | 0,00 | 0,00 | 0,00 | Rhaponticin |
| 73 | 122 | 0,00 | 0,00 | 0,00 | Sennoside A |
| 74 | 123 | 0,00 | 0,00 | 0,00 | Scopoletin |
| 75 | 125 | 0,00 | 0,00 | 0,00 | Naringenin |
| 76 | 137 | 15,84 | 14,69 | 4,90 | D-Amygdalin |
| 77 | 138 | 0,00 | 0,00 | 0,00 | Amantadine |
| 78 | 139 | 0,00 | 0,00 | 0,00 | (+)-Catechol hydrate |
| 79 | 140 | 9,12 | 2,46 | 0,82 | Quinic acid |
| 80 | 141 | 0,00 | 0,00 | 0,00 | m-Coumaric acid |
| 81 | 142 | 13,99 | 7,71 | 2,57 | 4-Hydroxy-3-methoxycinnamic acid |
| 82 | 145 | 0,00 | 0,00 | 0,00 | p-Coumaric acid |
| 83 | 146 | 0,00 | 0,00 | 0,00 | Theophylline |
| 84 | 147 | 0,00 | 0,00 | 0,00 | Curcumin |
| 85 | 148 | 0,00 | 0,00 | 0,00 | Isoquercitrin |
| 86 | 150 | 0,00 | 0,00 | 0,00 | Theobromine |
| 87 | 151 | 100,00 | 0,00 | 0,00 | Quercetin |
| 88 | 152 | 0,00 | 0,00 | 0,00 | Luteolin |

**TableS 2.** A list of descriptors used to train the machine learning-based QSAR model, excluding additional docking parameters.

| nAcid | ALogP | ALogp2 | AMR | apol |
| --- | --- | --- | --- | --- |
| naAromAtom | nAromBond | nAtom | nHeavyAtom | nH |
| nC | nN | nO | ATS0m | ATS1m |
| ATS2m | ATS3m | ATS4m | ATS5m | ATS6m |
| ATS7m | ATS8m | ATS0v | ATS1v | ATS2v |
| ATS3v | ATS4v | ATS5v | ATS6v | ATS7v |
| ATS8v | ATS0e | ATS1e | ATS2e | ATS3e |
| ATS4e | ATS5e | ATS6e | ATS7e | ATS8e |
| ATS0p | ATS1p | ATS2p | ATS3p | ATS4p |
| ATS5p | ATS6p | ATS7p | ATS8p | ATS0i |
| ATS1i | ATS2i | ATS3i | ATS4i | ATS5i |
| ATS6i | ATS7i | ATS8i | ATS0s | ATS1s |
| ATS2s | ATS3s | ATS4s | ATS5s | ATS6s |
| ATS7s | ATS8s | AATS0m | AATS1m | AATS2m |
| AATS3m | AATS4m | AATS5m | AATS6m | AATS7m |
| AATS8m | AATS0v | AATS1v | AATS2v | AATS3v |
| AATS4v | AATS5v | AATS6v | AATS7v | AATS8v |
| AATS0e | AATS1e | AATS2e | AATS3e | AATS4e |
| AATS5e | AATS6e | AATS7e | AATS8e | AATS0p |
| AATS1p | AATS2p | AATS3p | AATS4p | AATS5p |
| AATS6p | AATS7p | AATS8p | AATS0i | AATS1i |
| AATS2i | AATS3i | AATS4i | AATS5i | AATS6i |
| AATS7i | AATS8i | AATS0s | AATS1s | AATS2s |
| AATS3s | AATS4s | AATS5s | AATS6s | AATS7s |
| AATS8s | ATSC0c | ATSC1c | ATSC2c | ATSC3c |
| ATSC4c | ATSC5c | ATSC6c | ATSC7c | ATSC8c |
| ATSC0m | ATSC1m | ATSC2m | ATSC3m | ATSC4m |
| ATSC5m | ATSC6m | ATSC7m | ATSC8m | ATSC0v |
| ATSC1v | ATSC2v | ATSC3v | ATSC4v | ATSC5v |
| ATSC6v | ATSC7v | ATSC8v | ATSC0e | ATSC1e |
| ATSC2e | ATSC3e | ATSC4e | ATSC5e | ATSC6e |
| ATSC7e | ATSC8e | ATSC0p | ATSC1p | ATSC2p |
| ATSC3p | ATSC4p | ATSC5p | ATSC6p | ATSC7p |
| ATSC8p | ATSC0i | ATSC1i | ATSC2i | ATSC3i |
| ATSC4i | ATSC5i | ATSC6i | ATSC7i | ATSC8i |
| ATSC0s | ATSC1s | ATSC2s | ATSC3s | ATSC4s |
| ATSC5s | ATSC6s | ATSC7s | ATSC8s | AATSC0m |
| AATSC1m | AATSC2m | AATSC3m | AATSC4m | AATSC5m |
| AATSC6m | AATSC7m | AATSC8m | AATSC0v | AATSC1v |
| AATSC2v | AATSC3v | AATSC4v | AATSC5v | AATSC6v |
| AATSC7v | AATSC8v | AATSC1i | AATSC2i | AATSC3i |
| AATSC4i | AATSC5i | AATSC6i | AATSC7i | AATSC8i |
| AATSC0s | AATSC1s | AATSC2s | AATSC3s | AATSC4s |
| AATSC5s | AATSC6s | AATSC7s | AATSC8s | MATS1c |
| MATS2c | MATS3c | MATS4c | MATS5c | MATS6c |
| MATS7c | MATS8c | MATS3m | MATS4m | MATS5m |
| MATS6m | MATS7m | MATS8m | MATS2v | MATS3v |
| MATS4v | MATS5v | MATS6v | MATS7v | MATS8v |
| MATS2e | MATS3e | MATS4e | MATS5e | MATS6e |
| MATS7e | MATS8e | MATS1p | MATS2p | MATS3p |
| MATS5p | MATS6p | MATS7p | MATS8p | MATS1i |
| MATS2i | MATS3i | MATS4i | MATS5i | MATS6i |
| MATS7i | MATS8i | MATS2s | MATS3s | MATS4s |
| MATS5s | MATS6s | MATS7s | MATS8s | GATS1c |
| GATS2c | GATS3c | GATS4c | GATS5c | GATS6c |
| GATS7c | GATS8c | GATS1m | GATS2m | GATS3m |
| GATS4m | GATS5m | GATS6m | GATS7m | GATS8m |
| GATS1v | GATS2v | GATS3v | GATS4v | GATS5v |
| GATS6v | GATS7v | GATS8v | GATS1e | GATS2e |
| GATS3e | GATS4e | GATS5e | GATS6e | GATS7e |
| GATS8e | GATS1p | GATS2p | GATS3p | GATS4p |
| GATS5p | GATS6p | GATS7p | GATS8p | GATS1i |
| GATS2i | GATS3i | GATS4i | GATS5i | GATS6i |
| GATS7i | GATS8i | GATS1s | GATS2s | GATS3s |
| GATS4s | GATS5s | GATS6s | GATS7s | GATS8s |
| SpAbs_DzZ | SpMax_DzZ | SpDiam_DzZ | SpAD_DzZ | SpMAD_DzZ |
| EE_DzZ | SM1_DzZ | VE3_DzZ | VR1_DzZ | VR2_DzZ |
| VR3_DzZ | SpAbs_Dzm | SpMax_Dzm | SpDiam_Dzm | SpAD_Dzm |
| SpMAD_Dzm | EE_Dzm | SM1_Dzm | VE3_Dzm | VR1_Dzm |
| VR2_Dzm | VR3_Dzm | SpAbs_Dzv | SpMax_Dzv | SpDiam_Dzv |
| SpAD_Dzv | SpMAD_Dzv | EE_Dzv | SM1_Dzv | VE1_Dzv |
| VE3_Dzv | VR1_Dzv | VR2_Dzv | VR3_Dzv | SpAbs_Dze |
| SpMax_Dze | SpDiam_Dze | SpAD_Dze | SpMAD_Dze | EE_Dze |
| SM1_Dze | VE3_Dze | VR1_Dze | VR2_Dze | VR3_Dze |
| SpAbs_Dzp | SpMax_Dzp | SpDiam_Dzp | SpAD_Dzp | SpMAD_Dzp |
| EE_Dzp | SM1_Dzp | VE1_Dzp | VE3_Dzp | VR1_Dzp |
| VR2_Dzp | VR3_Dzp | SpAbs_Dzi | SpMax_Dzi | SpDiam_Dzi |
| SpAD_Dzi | SpMAD_Dzi | EE_Dzi | SM1_Dzi | VE3_Dzi |
| VR1_Dzi | VR2_Dzi | VR3_Dzi | SpAbs_Dzs | SpMax_Dzs |
| SpDiam_Dzs | SpAD_Dzs | SpMAD_Dzs | EE_Dzs | SM1_Dzs |
| VE1_Dzs | VE3_Dzs | VR1_Dzs | VR2_Dzs | VR3_Dzs |
| nBase | BCUTw-1h | BCUTp-1l | BCUTp-1h | nBonds |
| nBonds2 | nBondsS | nBondsS2 | nBondsS3 | nBondsD |
| nBondsT | nBondsM | bpol | SpMax2_Bhm | SpMax3_Bhm |
| SpMax4_Bhm | SpMax5_Bhm | SpMax6_Bhm | SpMax7_Bhm | SpMax8_Bhm |
| SpMin2_Bhm | SpMin3_Bhm | SpMin4_Bhm | SpMin5_Bhm | SpMin6_Bhm |
| SpMin7_Bhm | SpMin8_Bhm | SpMax2_Bhv | SpMax3_Bhv | SpMax4_Bhv |
| SpMax5_Bhv | SpMax6_Bhv | SpMax7_Bhv | SpMax8_Bhv | SpMin2_Bhv |
| SpMin3_Bhv | SpMin4_Bhv | SpMin5_Bhv | SpMin6_Bhv | SpMin7_Bhv |
| SpMin8_Bhv | SpMax2_Bhe | SpMax3_Bhe | SpMax4_Bhe | SpMax5_Bhe |
| SpMax6_Bhe | SpMax7_Bhe | SpMax8_Bhe | SpMin2_Bhe | SpMin3_Bhe |
| SpMin4_Bhe | SpMin5_Bhe | SpMin6_Bhe | SpMin7_Bhe | SpMin8_Bhe |
| SpMax2_Bhp | SpMax3_Bhp | SpMax4_Bhp | SpMax5_Bhp | SpMax6_Bhp |
| SpMax7_Bhp | SpMax8_Bhp | SpMin2_Bhp | SpMin3_Bhp | SpMin4_Bhp |
| SpMin5_Bhp | SpMin6_Bhp | SpMin7_Bhp | SpMin8_Bhp | SpMax2_Bhi |
| SpMax3_Bhi | SpMax4_Bhi | SpMax5_Bhi | SpMax6_Bhi | SpMax7_Bhi |
| SpMax8_Bhi | SpMin2_Bhi | SpMin3_Bhi | SpMin4_Bhi | SpMin5_Bhi |
| SpMin6_Bhi | SpMin7_Bhi | SpMin8_Bhi | SpMax1_Bhs | SpMax2_Bhs |
| SpMax3_Bhs | SpMax4_Bhs | SpMax5_Bhs | SpMax6_Bhs | SpMax7_Bhs |
| SpMax8_Bhs | SpMin2_Bhs | SpMin3_Bhs | SpMin4_Bhs | SpMin5_Bhs |
| SpMin6_Bhs | SpMin7_Bhs | SpMin8_Bhs | C1SP2 | C2SP2 |
| C3SP2 | C1SP3 | C2SP3 | C3SP3 | C4SP3 |
| SCH-6 | SCH-7 | VCH-6 | VCH-7 | SC-3 |
| SC-5 | VC-3 | VC-5 | SPC-4 | SPC-5 |
| SPC-6 | VPC-4 | VPC-5 | VPC-6 | SP-0 |
| SP-1 | SP-2 | SP-3 | SP-4 | SP-5 |
| SP-6 | SP-7 | VP-0 | VP-1 | VP-2 |
| VP-3 | VP-4 | VP-5 | VP-6 | VP-7 |
| Sv | Sse | Spe | Sare | Sp |
| Si | CrippenLogP | CrippenMR | SpMax_Dt | SpDiam_Dt |
| SpAD_Dt | SpMAD_Dt | EE_Dt | VE3_Dt | VR1_Dt |
| VR2_Dt | VR3_Dt | ECCEN | nHBd | nHBa |
| nwHBa | nHBint2 | nHBint3 | nHBint4 | nHBint5 |
| nHBint6 | nHBint7 | nHBint8 | nHBint9 | nHBint10 |
| nHsOH | nHsNH2 | nHssNH | nHaaNH | nHdCH2 |
| nHdsCH | nHaaCH | nHCsats | nHCsatu | nHAvin |
| nHother | nsCH3 | nssCH2 | nsssCH | ndssC |
| naasC | naaaC | nssssC | naaN | nsssN |
| naasN | ndO | nssO | naaO | SHBd |
| SHBa | SwHBa | SHBint2 | SHBint3 | SHBint4 |
| SHBint5 | SHBint6 | SHBint7 | SHBint8 | SHBint9 |
| SHBint10 | SHsOH | SHdsCH | SHaaCH | SHCsats |
| SHCsatu | SHAvin | SHother | SsCH3 | SdCH2 |
| SssCH2 | SdsCH | SaaCH | SsssCH | StsC |
| SdssC | SaasC | SaaaC | SssssC | SsNH2 |
| SssNH | SaaNH | StN | SaaN | SsssN |
| SaasN | SsOH | SdO | SssO | SaaO |
| minHBd | minHBa | minwHBa | minHBint2 | minHBint3 |
| minHBint4 | minHBint5 | minHBint6 | minHBint7 | minHBint8 |
| minHBint9 | minHBint10 | minHsOH | minHdsCH | minHaaCH |
| minHCsats | minHCsatu | minHAvin | minHother | minsCH3 |
| minssCH2 | mindsCH | minaaCH | minsssCH | mindssC |
| minaasC | minaaaC | minssssC | minsssN | minsOH |
| mindO | minssO | maxHBd | maxHBa | maxwHBa |
| maxHBint2 | maxHBint3 | maxHBint4 | maxHBint5 | maxHBint6 |
| maxHBint7 | maxHBint8 | maxHBint9 | maxHBint10 | maxHsOH |
| maxHdsCH | maxHaaCH | maxHCsats | maxHCsatu | maxHAvin |
| maxHother | maxsCH3 | maxssCH2 | maxdsCH | maxaaCH |
| maxsssCH | maxdssC | maxaasC | maxaaaC | maxsssN |
| maxsOH | maxdO | maxssO | sumI | meanI |
| hmax | gmax | hmin | gmin | LipoaffinityIndex |
| MAXDN | MAXDP | DELS | ETA_Alpha | ETA_Beta |
| ETA_BetaP | ETA_Beta_s | ETA_Beta_ns | ETA_BetaP_ns | ETA_dBeta |
| ETA_dBetaP | ETA_Beta_ns_d | ETA_Eta | ETA_EtaP | ETA_Eta_R |
| ETA_Eta_F | ETA_EtaP_F | ETA_Eta_L | ETA_Eta_R_L | ETA_Eta_F_L |
| ETA_Eta_B | ETA_Eta_B_RC | fragC | nHBAcc | nHBAcc2 |
| nHBAcc3 | nHBAcc_Lipinski | nHBDon_Lipinski | HybRatio | IC0 |
| IC1 | IC2 | IC3 | IC4 | IC5 |
| TIC0 | TIC1 | TIC2 | TIC3 | TIC4 |
| TIC5 | CIC0 | CIC1 | CIC2 | CIC3 |
| CIC4 | CIC5 | MIC0 | MIC1 | MIC2 |
| MIC3 | MIC4 | MIC5 | ZMIC0 | ZMIC1 |
| ZMIC2 | ZMIC3 | ZMIC4 | ZMIC5 | Kier1 |
| Kier2 | Kier3 | nAtomLC | nAtomP | nAtomLAC |
| MLogP | McGowan_Volume | MDEC-11 | MDEC-12 | MDEC-13 |
| MDEC-14 | MDEC-22 | MDEC-23 | MDEC-24 | MDEC-33 |
| MDEC-34 | MDEC-44 | MDEO-11 | MDEO-12 | MDEO-22 |
| MDEN-23 | MDEN-33 | MLFER_A | MLFER_BH | MLFER_BO |
| MLFER_S | MLFER_E | MLFER_L | MPC2 | MPC3 |
| MPC4 | MPC5 | MPC6 | MPC7 | MPC8 |
| MPC9 | MPC10 | TPC | piPC1 | piPC2 |
| piPC3 | piPC4 | piPC5 | piPC6 | piPC7 |
| piPC8 | piPC9 | piPC10 | TpiPC | R_TpiPCTPC |
| nRing | n3Ring | n5Ring | n6Ring | nFRing |
| nF6Ring | nF7Ring | nF8Ring | nF9Ring | nF10Ring |
| nF12Ring | nFG12Ring | nTRing | nT6Ring | nHeteroRing |
| n5HeteroRing | n6HeteroRing | nF6HeteroRing | nF8HeteroRing | nF10HeteroRing |
| nFG12HeteroRing | nT6HeteroRing | nRotB | nRotBt | RotBtFrac |
| LipinskiFailures | topoRadius | topoDiameter | topoShape | GGI1 |
| GGI2 | GGI3 | GGI4 | GGI5 | GGI6 |
| GGI7 | GGI8 | GGI9 | GGI10 | SpMax_D |
| SpDiam_D | SpAD_D | SpMAD_D | EE_D | VE3_D |
| VR1_D | VR2_D | VR3_D | TopoPSA | VABC |
| VAdjMat | MWC2 | MWC3 | MWC4 | MWC5 |
| MWC6 | MWC7 | MWC8 | MWC9 | MWC10 |
| TWC | SRW2 | SRW3 | SRW4 | SRW5 |
| SRW6 | SRW7 | SRW8 | SRW9 | SRW10 |
| TSRW | MW | AMW | WTPT-1 | WTPT-3 |
| WTPT-4 | WTPT-5 | WPATH | WPOL | XLogP |
| Zagreb |  |  |  |  |

**TableS 3.** Random Forest models evaluation.

| Exp No | Taregeted_test_compounds | | | Docking_Concensus | | Model_Performance | |
| --- | --- | --- | --- | --- | --- | --- | --- |
|  | 30 | 35 | 87 | Yes | No | Accuracy | ROC_Score |
| **1*** | **/** | **/** | **-** | **-** | **/** | **0.69** | **0.67** |
| 2 | / | / | - | - | / | 0.60 | 0.59 |
| 3 | / | / | - | - | / | 0.60 | 0.59 |
| 4 | / | / | - | - | / | 0.65 | 0.63 |
| 5 | / | / | - | - | / | 0.65 | 0.63 |
| 6 | / | / | - | - | / | 0.60 | 0.59 |
| 7 | / | / | - | - | / | 0.69 | 0.67 |
| 8 | / | / | - | - | / | 0.60 | 0.59 |
| 9 | / | / | - | - | / | 0.60 | 0.59 |
| 10 | / | / | - | / | - | 0.60 | 0.59 |
| 11 | / | / | - | - | / | 0.60 | 0.59 |
| 12 | / | / | - | - | / | 0.60 | 0.59 |
| 13 | / | / | - | - | / | 0.60 | 0.59 |
| 14 | / | / | - | - | / | 0.60 | 0.59 |
| 15 | / | / | - | - | / | 0.56 | 0.56 |
| 16 | / | / | - | - | / | 0.56 | 0.56 |
| 17 | / | / | - | / | - | 0.56 | 0.56 |
| 18 | / | / | - | - | / | 0.65 | 0.63 |
| 19 | / | / | - | - | / | 0.60 | 0.59 |
| 20 | / | / | - | - | / | 0.65 | 0.63 |
| 21 | / | / | - | - | / | 0.60 | 0.59 |
| **22*** | **/** | **/** | **-** | **/** | **-** | **0.65** | **0.63** |
| 23 | / | / | - | - | / | 0.60 | 0.59 |
| 24 | / | / | - | - | / | 0.56 | 0.56 |
| 25 | / | / | - | - | / | 0.60 | 0.59 |
| 26 | / | / | - | / | - | 0.60 | 0.59 |
| 27 | / | / | - | - | / | 0.56 | 0.56 |
| 28 | / | / | - | / | - | 0.56 | 0.56 |
| 29 | / | / | - | - | / | 0.56 | 0.56 |
| 30 | / | / | - | - | / | 0.60 | 0.59 |

Grey highlights the best models with and without consensus docking. The selected models are bold and designated with an asteroid (*). Models 1 and 7 exhibit the same performance level for the models without consensus docking. However, model 1 is selected because its important features correlate better with the best model with consensus docking (model 22).

**TableS 4.** Logistic regression models evaluation.

| Exp No | Taregeted_test_compounds | | | Docking_Concensus | | Model_Performance | |
| --- | --- | --- | --- | --- | --- | --- | --- |
|  | 30 | 35 | 87 | Yes | No | Accuracy | ROC_Score |
| **1*** | **/** | **-** | **-** | **-** | **/** | **0.56** | **0.56** |
| 2 | / | - | - | - | / | 0.52 | 0.52 |
| 3 | / | - | - | - | / | 0.52 | 0.52 |
| 4 | / | - | - | - | / | 0.52 | 0.52 |
| 5 | / | - | - | - | / | 0.52 | 0.52 |
| **6*** | **/** | **-** | **-** | **/** | **-** | **0.52** | **0.50** |
| 7 | / | - | - | - | / | 0.52 | 0.52 |
| 8 | / | - | - | - | / | 0.56 | 0.56 |
| 9 | / | - | - | - | / | 0.52 | 0.52 |
| 10 | / | - | - | - | / | 0.52 | 0.52 |
| 11 | / | - | - | - | / | 0.52 | 0.52 |
| 12 | / | - | - | - | / | 0.52 | 0.52 |
| 13 | / | - | - | - | / | 0.52 | 0.52 |
| 14 | / | - | - | - | / | 0.52 | 0.52 |
| 15 | / | - | - | - | / | 0.52 | 0.52 |
| 16 | / | - | - | - | / | 0.52 | 0.52 |
| 17 | / | - | - | - | / | 0.52 | 0.52 |
| 18 | / | - | - | - | / | 0.56 | 0.56 |
| 19 | / | - | - | - | / | 0.52 | 0.52 |
| 20 | / | - | - | - | / | 0.52 | 0.52 |
| 21 | / | - | - | - | / | 0.52 | 0.52 |
| 22 | / | - | - | - | / | 0.52 | 0.52 |
| 23 | / | - | - | / | - | 0.52 | 0.50 |
| 24 | / | - | - | - | / | 0.52 | 0.52 |
| 25 | / | - | - | - | / | 0.52 | 0.52 |
| 26 | / | - | - | - | / | 0.52 | 0.52 |
| 27 | / | - | - | / | - | 0.52 | 0.50 |
| 28 | / | - | - | - | / | 0.52 | 0.52 |
| 29 | / | - | - | - | / | 0.52 | 0.52 |
| 30 | / | - | - | / | - | 0.52 | 0.50 |

Grey highlights the best models with and without consensus docking. The selected models are bold and designated with asteroid (*).
